# Paternal dietary ratio of n-6: n-3 polyunsaturated fatty acids programs offspring leptin expression and gene imprinting in mice

**DOI:** 10.3389/fnut.2022.1043876

**Published:** 2022-12-23

**Authors:** Qiaoyu Shi, Xuanyi Liu, Xiuqin Fan, Rui Wang, Kemin Qi

**Affiliations:** Laboratory of Nutrition and Development, Key Laboratory of Major Diseases in Children, National Center for Children’s Health, Ministry of Education, Beijing Pediatric Research Institute, Beijing Children’s Hospital, Capital Medical University, Beijing, China

**Keywords:** gene imprinting, paternal programming, n-3 fatty acids, leptin, offspring

## Abstract

**Background:**

This study determined the effects of the paternal dietary ratio of n-6: n-3 polyunsaturated fatty acids (PUFAs) on leptin expression in the offspring and associated gene imprinting in a mouse model.

**Methods:**

Three- to four-week-old male C57BL/6J mice (F0) were fed an n-3 PUFA-deficient (n-3 D) diet, a diet with normal n-3 PUFA content (n-3 N; n-6: n-3 = 4.3:1), or a diet with a high n-3 PUFA content (n-3 H; n-6: n-3 = 1.5:1) for 8 weeks. Two subsequent generations were generated by mating F0 and F1 male mice with 10-week-old virgin female C57 BL/6J mice, to produce F1 and F2 offspring.

**Results:**

Compared to the paternal n-3 D diet, paternal n-3 N and n-3 H diets reduced adipose mRNA expression of leptin (Lep) and its plasma concentrations in juvenile F1 male and female offspring, and adult F1 male and F2 female offspring, with upregulated Lep receptor mRNA expression in the hypothalamus. Meanwhile, paternal n-3 N and n-3 H diets altered the expression of the imprinted genes *H19*, *Igf2*, *Igf2r*, *Plagl1*, *Cdkn1c*, *Kcnq1ot1*, *Peg3*, and *Grb10* in the adipose tissue of juvenile and adult F1 males, with almost no effects on F1 females, while more effects were observed in the adult F2 females than F2 males. Principal component analysis verified that *Plagl1*, *Cdkn1c*, and *Kcnq1ot1* contributed the most to variation in adipose tissue expression in all offspring. Some of these genes (*Plagl1*, *Cdkn1c*, *Kcnq1ot1*, *Peg3*, and *Grb10*) were altered by the paternal n-3 N and n-3 H diets in the F1 and F2 generation testes as well. Furthermore, adipose Lep expression was positively correlated with expressions of *H19*, *Igf2r*, *Plagl1*, and *Kcnq1ot1* in juvenile F1 males and females, negatively correlated with the *Kcnq1ot1* expression in adult F1 males, and positively correlated with the *Plagl1* expression in adult F2 females.

**Conclusion:**

These data imply that paternal *Plagl1*, *Cdkn1c*, and *Kcnq1ot1* might be part of the pathways involved in offspring leptin programming. Therefore, a lower ratio of n-6: n-3 PUFAs, with higher intake of n-3 PUFAs in paternal pre-conception, may help maintain the offspring’s optimal leptin pattern in a sex-specific manner through multiple generations, and thereby, be beneficial for the offspring’s long-term health.

## 1 Introduction

The fetal programming and developmental origins of health and disease hypotheses suggest that environmental factors in early life can alter the phenotype and health of offspring in later life (1, 2). Over the past decade, increasing evidence has shown that not only the maternal nutritional status, but also the pre-conceptional diet modifications in fathers, whether it be excess or restriction of nutrients, lead to negative pregnancy outcomes, metabolic changes in the progeny, and/or long-term consequences (3–5). Both maternal and paternal effects can be carried beyond the F1 generations and transferred across multiple generations, from the parents (F0) to the grandoffspring (F2/F3 and beyond), and are thus termed transgenerational effects (6). Meanwhile, sex-specific, male-line transgenerational responses to paternal nutrition and the environment have been observed in populations and animal models. For example, early paternal smoking has been found to be associated with greater body mass index in sons, but not in daughters, and the mortality risk ratios of grandsons are influenced by the diet of their paternal grandfather, while those of the granddaughters are influenced by the diet of their paternal grandmother (7, 8). Kaati et al. found that during the slow growth period, the paternal grandmother’s exposure to famine was associated with lower cardiovascular causes of death, whereas the paternal grandfather’s exposure to a surfeit of food was related to increased diabetes mortality in grandchildren (9). Thereafter, multigenerational studies have provided evidence that transgenerational inheritance can persist in a lineage-specific (to F2s, *via* either the paternal or maternal lineage) and/or sex-specific (to only male or female F2s) fashion, through multiple generations (10–14).

Identification of the obesity gene-encoded leptin in 1994 and characterization of its function as a major appetite suppressant are landmarks in obesity, endocrinology, and metabolism research (15, 16). Leptin is primarily synthesized and secreted by the adipose tissue, with the stomach, skeletal muscle, and liver also contributing to small amounts. It functions as an extensive modulator in the metabolism of various systems and inflammation (17), beyond its role in maintaining energy homeostasis and body fat at levels required for both the central nervous and peripheral systems, by acting on the leptin receptor (18, 19). At low or moderate levels, leptin supports physiological roles, but chronically higher doses exhibit detrimental effects on various systems (17). In most cases, circulating leptin levels are elevated in obese subjects, who are believed to develop leptin resistance, which is characterized by the reduced ability of leptin to suppress appetite and weight gain (19, 20). Recently, it has been reported that maintaining lower levels of leptin expression in animal adulthood protects against high-fat-induced obesity and associated metabolic disorders, and increases sensitivity to exogenous leptin (21, 22). In addition, in the early stages of life, maintenance of the optimal expression pattern and trajectory of leptin and leptin sensitivity is of vital importance for the development of central and peripheral systems involved in energy homeostasis and brain development, and thus, is helpful for the prevention of chronic metabolic and neurodegenerative diseases in later life (23–28).

Several food components, such as phenols, peptides, vitamins, and n-3 polyunsaturated fatty acids (n-3 PUFAs), can improve leptin sensitivity, by upregulating or downregulating leptin signaling molecules and reducing inflammation (29). Maternal supplementation with n-3 PUFAs during pregnancy and the balance between n-6 and n-3 PUFAs (lower ratio of n-6: n-3 PUFAs) generally reduces leptin expression and its circulating level, through epigenetic modification of the gene promoter in young and adult offspring of both animals and humans, which may be beneficial for later health; however, conclusions in this respect have not been consistent (30–36). It has been demonstrated that mammalian spermatozoa are characterized by a high proportion of PUFAs, particularly docosahexaenoic acid (DHA, C22: 6n-3), and that increased intake of n-3 PUFAs in men, either as supplements or from foods, appears to have a positive effect on spermatogenesis, with higher sperm motility, normal morphology, and concentration (37–39). However, little is known about the impact of paternal n-3 PUFA status on the pre- and postnatal growth and development of offspring and their later health.

Epigenetic modifications of target genes in somatic or germline cells are key pathways that mediate the effects of parental nutrition on offspring development and health in intergenerational and transgenerational transmission (3–5, 40). Sperm epigenetic status (DNA methylation, histone modifications, and RNA methylation) and microRNA content are modulated by poor paternal peri-conceptional nutrition, which are potential signals that program offspring health and initiate the transmission of obesity, glucose intolerance, impaired vascular function, and non-alcoholic fatty liver disease to future generations (12, 41, 42). Gene imprinting, a process that reversibly marks one of the two homologous loci, chromosome/chromosomal sets, and functional non-equivalence of gene expression, plays an important role in modulating fetal-placental growth and resource acquisition, postnatal growth and energy homeostasis, and transgenerational inheritance (43–45). Abnormalities in imprinting genes, caused by mutations, deletions, and epigenetic aberrations, can result in the loss of imprinting and imprinting disorders such as complex syndromes of neurodevelopmental disabilities, cognitive problems, and metabolic diseases (46). In addition, imprinted gene products act at multiple levels in the adipose-hypothalamic axis, to modulate set-points of energy homeostasis, by altering leptin expression and the sensitivity of neuroendocrine pathways to leptin (43).

With respect to the association between gene imprinting and leptin, it has been reported that the products of *Mest* and *Grb10* promote leptin production or leptin signaling, whereas those of *Cdkn1c*, *Dlk1*, *H19*, *Peg3*, and *Magel2* can suppress leptin production or its actions (47–51). Specifically, *Mest*-knockout mice show lighter body weight with reduced adiposity (48), whereas diet-induced or genetically obese mice display enhanced expression of *Mest* in the white adipose tissue, and ectopic expression of *Mest* increases the expression of leptin and other adipose markers such as peroxisome proliferator-activated receptor γ (PPARγ), CCAAT/enhancer binding protein α and adipocyte fatty acid binding protein 2, with enlarged adipocytes (52). *Grb10* encodes an intracellular signaling adaptor protein that restricts fetal growth and is permissive of adipose deposition in adulthood, with increased leptin levels (53, 54). Moreover, offspring *Grb10* suppresses the development of lean mass, whereas the offspring fat mass is promoted by *Grb10* expressed in the mother and acts on postnatal nutrient supply, jointly promoting optimal offspring body proportions (49). In contrast, the lack of *Peg3*, which encodes a zinc finger protein, leads to growth retardation in early life and increases adiposity in adult males, with increased leptin expression (47, 55). *Dlk1*, known to play a crucial role in adipose homeostasis, promotes fetal growth and restricts adipose deposition and leptin expression in adults (53, 56–58). Mice lacking *Dlk1* display pre- and postnatal growth retardation, followed by a catching up in body weight, at approximately 90 d for females and 65 d for males, with increased leptin expression (53, 56, 57), whereas overexpression of *Dlk1* causes a shift in the metabolic mode of the organism toward peripheral lipid oxidation and away from lipid storage, with downregulation of leptin expression (58). Similarly, *Magel2*-null pups endure growth retardation after birth, equalize their mean weight to wild-type levels at a young age, and subsequently gain more weight than their wild-type littermates in adulthood, with a higher plasma leptin level (59), due to decline in leptin sensitivity (60). Although their contribution to leptin expression has not been reported, *Plagl1*, *Cdkn1c*, and *Kcnq1ot1* have been found to be involved in adiposity and obesity (61–68), implying that they may impact leptin expression in the adipose tissue. Beyond its association with metabolic, genetic, and neoplastic illnesses through multiple pathways (61), *Plagl1* has been found to be highly expressed in the white adipose tissue of adult rats, as compared to the other organs and tissues, and has a positive correlation with PPARγ and fatty acid binding protein 4 (62). Increased expression of *Cdkn1c* causes the child growth-restriction disorders, Silver–Russell and IMAGe syndromes, while loss of its function causes the overgrowth disorder Beckwith–Wiedemann syndrome (63, 64). Using a transgenic mouse model, Vega-Benedetti et al. found that overexpression of *Cdkn1c* during development protects against age- and diet-induced obesity, by promoting brown adipose tissue formation, with reduced adiposity (65, 66). The expression of *Kcnq1ot1* is downregulated in the mouse cartilage tissue of osteoarthritis and islets in high-fat-induced obesity, and knockdown of *Kcnq1ot1 in vivo* reduces glucose tolerance and insulin secretion (67, 68).

Therefore, it is of great interest to determine whether the dietary ratio of n-6: n-3 PUFAs in paternal pre-conception can affect offspring leptin expression and associated growth and development, with transgenerational transmission. In the present study, using a mouse model that received feeding intervention, we investigated the effects of pre-conception n-3 PUFA supplementation in the father (F0) on leptin expression in the offspring (F1 and F2) and associated gene imprinting.

## 2 Materials and methods

### 2.1 Animals and diets

All experiments complied with the ARRIVE guidelines, and all procedures were conducted in accordance with the Animals (Scientific Procedures) 1986 Act (UK) (amended 2013) and the Guide for the Care and Use of Laboratory Animals in China and approved by the Ethics Committee of the National Institute of Occupational Health and Poison Control, China CDC (Beijing, China) (approval no. EAWE-2021-06). Three- to four-week-old male C57BL/6J mice were purchased from Beijing Si Pei Fu Biotechnology Co., Ltd. (Beijing, China) and housed at the animal facilities of the National Institute of Occupational Health and Poison Control, China CDC, under specific pathogen-free grade conditions. These founder (F0) mice were provided one of three diets, an n-3 PUFA-deficient (n-3 D) diet, a diet with normal n-3 PUFA content (n-3 N) and an n-6: n-3 ratio of 4.3:1, or a diet with high n-3 PUFA content (n-3 H) and an n-6: n-3 ratio of 1.5:1, for 8 weeks. The diets were designed by modifying the fat type to generate different n-3 PUFA contents, as previously described (69). The diet formula for the fat content and fatty acid composition is shown in Table 1. All diets were manufactured by Beijing Huafukang Bioscience Co., Ltd. (Beijing, China), and stored at −20°C before use.

**TABLE 1:** The diet formula with fat contents and fatty acid compositions.

	n-3 D	n-3 N	n-3 H
Energy (kcal/g)	3.9	3.9	3.9
Ingredient	g	g	g
Casein	200	200	200
Cystine	3	3	3
Corn starch	397	397	397
Maltodextrin	132	132	132
Sucrose	100	100	100
Cellulose	50	50	50
Lard oil	22	22	22
Sunflower oil	48	37	22
Flaxseed oil	0	7	17
Fish oil	0	4	9
Mineral mix	35	35	35
Vitamin mix	10	10	10
Choline	2.5	2.5	2.5
Antioxidant TBHQ	0.014	0.014	0.014
Total	1,000	1,000	1,000
**Fatty acid (% of total fatty acids)**			
Saturated	33.19	33.85	33.53
Mono-unsaturated	27.29	25.37	25.07
Total n-6 PUFAs	38.70	33.14	24.95
18:2 n-6 (LA)	38.04	32.29	24.23
18:3 n-6 (GLA)	0.41	0.47	0.34
20:3 n-6 (DGLA)	0.04	0.07	0.05
20:4 n-6 (AA)	0.14	0.16	0.19
22:2 n-6 (DDA)	0.05	0.09	0.07
22:4 n-6 (ADA)	0.02	0.06	0.06
22:5 n-6 (OA)	–	–	0.01
Total n-3 PUFAs	0.82	7.64	16.45
18:3 n-3 (ALA)	0.28	5.74	13.54
18:4 n-3 (STA)	0.32	0.46	0.32
20:3 n-3 (EA)	0.22	0.38	0.28
20:5 n-3 (EPA)	–	0.80	1.60
22:5 n-3 (DPA)	–	0.06	0.11
22:6 n-3 (DHA)	–	0.20	0.60
n-6: n-3 PUFAs	47.2:1	4.3:1	1.5:1

n-3 PUFAs, polyunsaturated fatty acids; LA, linoleic acid; GLA, γ-linoleic acid; DGLA, dihomo-γ-linolenic acid; AA, arachidonic acid; DDA, decanedicarboxylic acid; ADA, adrenic acid; OA, osbond acid; ALA, α-linolenic acid; STA, stearidonicacid; EA, eicosatrienoic acid; EPA, eicosapentaenoic acid; DPA, docosapentaenoci acid; DHA, docosahexaenoic acid; n-3 D, n-3 PUFA-deficient diet; n-3 N, normal n-3 PUFA content diet; n-3 H, high n-3 PUFA content diet.

In case of the n-3 D diet, lard oil and sunflower oil were added to make the diet n-3 PUFA-deficient, with an n-6: n-3 PUFA ratio of 47.2:1. In case of the n-3 N and n-3 H diets, flaxseed and fish oils (the main sources of n-3 PUFAs) were combined with sunflower oil to yield two different n-6: n-3 PUFA ratios of 4.3:1 and 1.5:1, respectively, which represent the recommended ratio (4–10:1) and ratio in our ancestors’ diet (1–2:1), respectively, containing both longer-chain n-3 PUFAs, such as eicosapentaenoic acid (EPA; C20:5n-3) and DHA, and their precursor α-linolenic acid (ALA, C18:3n-3) (70, 71). The n-3 N and n-3 H diets contained ALA longer-chain n-3 PUFAs, at concentrations of 7.64 and 16.45% of the total fatty acids, respectively, equivalent to 1.20 and 2.59% of the total energy (TE), respectively. The dose of n-3 PUFAs is reasonably achievable in humans, within the range of minimum and safe values. The World Health Organization recommends that the total n-3 PUFA intake can range between 0.5 and 2% TE, whereas the minimum dietary requirement of ALA (>0.5% TE) for adults prevents deficiency symptoms and a higher value of 2% TE (ALA) plus EPA and DHA (0.250–2.0 g) can be part of a healthy diet. Reference values in Australia, New Zealand, and US (by the Food and Drug Administration) for the upper and safe intake of EPA plus DHA have been set at 3 g/d (72). In Eskimos, the average intake of n-3 PUFAs obtained mostly from fish oils is approximately 5% of the TE (73).

### 2.2 Animal procedure

**FIGURE 1:**
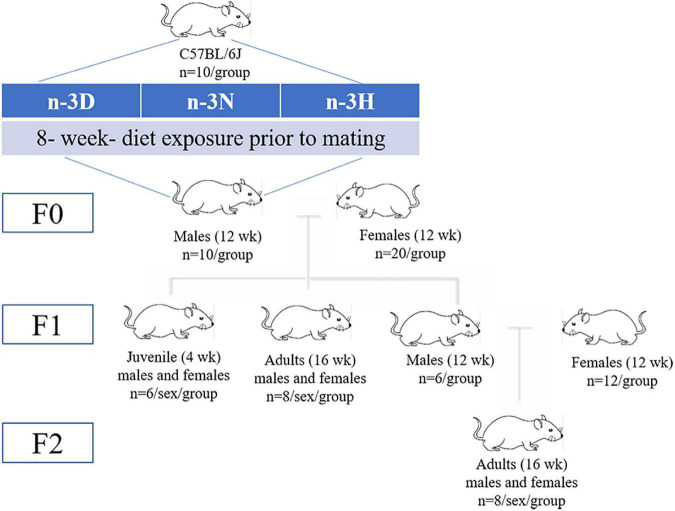
Schematic overview of the study design. Founder (F0) male mice were allocated either an n-3 polyunsaturated fatty acid (n-3 PUFA)-deficient (n-3 D) diet, a diet with normal n-3 PUFA content (n-3 N with an n-6: n-3 PUFA ratio at 4.3:1), or a diet with a high n-3 PUFA content (n-3 H with an n-6: n-3 PUFA ratio of 1.5:1) for 8 weeks. After 8 weeks of feeding intervention, the F0 mice were mated with 12-week-old virgin female normal-weight naturally cycling C57BL/6J mice (1 male for 2 females per cage), to produce F1 offspring. For production of the F2 generation, 12-week-old F1 males (each from separate litters) were mated naturally to 12-week-old virgin female C57BL/6 mice.

A diagrammatic representation of the study design and offspring generation is provided in Figure 1. After 8 weeks of feeding intervention, F0 mice (*n* = 10 per group) were mated with 12-week-old virgin female normal-weight naturally cycling C57BL/6J mice (one male for two females per cage), to produce F1 offspring. For production of the F2 generation, 12-week-old F1 males (*n* = 6 per group; each from separate litters) were mated naturally with 12-week-old virgin female C57BL/6 mice. In the case of males in which an epigenetic change is induced, the founder (F0) and his germline (future F1) are exposed; F1 is thus considered intergenerational, and F2 and subsequent generations are considered transgenerational (74). Therefore, in this study, the F2 generation was used to identify evidence of transgenerational inheritance. The mating female mice were fed AIN-93G diet (H10293G) throughout gestation and the 3 weeks of lactation, whereas all F1 and F2 offspring received the AIN-93M diet (H10293M) (Beijing Huafukang Bioscience Co., Ltd.) after weaning and water *ad libitum* throughout the study. At the end of the experiments, using intraperitoneal injection of avertin (2,2,2-tribromoethanol) (T-4840-2, Aldrich Chemical, Co., Inc., Milwaukee, USA) (125 mg/kg) for anesthesia followed by decapitation, F0 male mice after mating, F1 offspring at either 4 weeks (juvenile; *n* = 6 in each group) or 16 weeks (adult; *n* = 8 in each group) of age, and F2 offspring (*n* = 8 in each group) at 16 weeks of age were sacrificed. Each of the F1 or F2 mice in each diet group were from separate litters, to avoid being born from the same father. Blood samples were obtained *via* heart puncture, mixed with EDTA as an anticoagulant, and centrifuged at 3,000 rpm (4°C for 15 min), following which the plasma was aliquoted and stored at –80°C. Two cauda epidydimuses were collected from each F0 male mouse, cut three times, placed in a 1.5 mL eppendorf tube with 1 mL of Whittens-HEPES media (1 mM chlortetracycline, 20 mM Tris, 130 mM NaCl, and 5 mM cysteine, pH 7.2–7.4), and incubated at room temperature for 30 min (to allow the sperm to swim out). Sperms were centrifuged 300 × *g* for 5 min and washed twice with Whittens-HEPES medium. For the sperm count, a solution containing sperm was placed in the chamber of a Neubauer hemocytometer (depth: 0.100 mm; area: 0.0025 mm^2^), and the cells were counted under a light microscope (CX41RF, Olympus Corporation, Tokyo, Japan), at 400 × magnification. Two hemocytometers were used per animal, and 20 squares from a total of 100 squares were measured. After sacrifice, the epididymal fat, testis, and hypothalamus were immediately dissected free of the surrounding tissue, removed, frozen in liquid N_2_, and then transferred to -80°C until further use.

### 2.3 Estimation of plasma parameters

Mouse plasma leptin concentrations were estimated using the Mouse/Rat Leptin Quantikine^®^ ELISA Kit (MOB00B, R&D Systems, Minneapolis, MN, USA). The minimum detectable dose of mouse and rat leptin was in the range of 1.28–5.56 pg/mL. Recombinant human leptin cross-reacts by approximately 0.09% and interferes at concentrations > 10 ng/mL. Concentrations of plasma triglyceride and total cholesterol were assayed using the enzymatic procedures, gliseril phospo para amino phenazone and cholesterol oxidase p-aminophenol, respectively, with commercial kits (CH0101151 and CH0101152, Sichuan Maccura Science Tech. Co., Ltd., Chengdu, China). Plasma insulin concentrations were determined using the Mouse Insulin ELISA Kit (H203-1-2), while plasma glucose concentrations were tested using glucose oxidase method, with the GLU Kit (A154-1-1) (NanJing JianCheng Bioengineering Institute, Nanjing, China).

### 2.4 Fatty acid determination

The fatty acids in the F0 testis tissue were analyzed using gas chromatography, on a 6890N GC System (Agilent Technologies, Inc., Santa Clara, CA, USA) equipped with a flame ionization detector and injector, using the fatty acid methyl esters (FAMEs) method, as previously described (69, 75). The separation of FAMEs was conducted on an high resolution gas chromatography column (100 m 0.25 mm id 0.2 μm DB, P/N 112-88A7, HP-88) (Agilent Technologies, Inc.). The tissue fatty acid data were expressed as the percent (%) (wt/wt) of total fatty acids.

### 2.5 Analysis of mRNA expression

Total RNA extraction was performed using 80 mg of epididymal fat or 20 mg of testis or hypothalamus, with the RNAiso Plus Kit (9109, Takara Bio. Inc., Kusatsu Shiga, Japan), and then reverse transcribed to cDNA using the RT Kit (AT341-02, TransGen Biotech Co., Ltd., Beijing, China), according to the manufacturer’s instructions. The mRNA expression of targeted genes, including adipokines, leptin (Lep), adiponectin (Adipoq), and resistin (Retn), leptin receptor (Lepr), growth- and development-associated imprinted genes (*H19*, *Igf2*, *Igf2r*, *Plagl1*, *Cdkn1c*, *Kcnq1ot1*, *Mest*, *Peg3*, *Dlk1*, *Grb10*, and *Magel2*), and epigenetically modifying enzymes, DNA methyltransferase (Dnmts; Dnmt1, Dnmt2, Dnmt3a, Dnmt3b, and Dnmt3l) and histone deacetylases (Hdacs; Hdac1, Hdac2, Hdac3, Hdac6, and Hdac9), was measured using real-time qPCR with a CFX96 Touch™ Real-Time PCR Detection System (Bio-Rad Laboratories, Inc., Hercules, CA, USA) and the Top Green qPCR SuperMix (AQ131-04, TransGen Biotech Co., Ltd.). Ribosomal protein large P0 (Rplp0), or glyceraldehyde-3-phosphate dehydrogenase (Gapdh) was used as the internal control. The expression levels of the targeted genes were normalized to those of the internal reference gene using the 2^–ΔCT^ method. The designed oligonucleotide primers for the targeted genes were verified using Primer-Blast,^1^ synthesized by Sangon Biotech (Shanghai) Co., Ltd. (Shanghai, China), as indicated in Supplementary Table 1.

### 2.6 Bisulfite conversion and sequencing

DNA methylation in the Lep promoter and differentially methylated regions (DMRs) of imprinted genes were analyzed using bisulfite sequencing, according to our previous method (35). Genomic DNA was isolated and purified from epididymal fat using an Animal Tissue DNA Kit (Simgen Biotechnology Co., Ltd., Hangzhou, China), while bisulfite modification was performed using an EZ DNA Methylation™ Kit (D5002, Zymo Research, Orange County, CA, USA). The converted DNA was amplified using nested PCR on a ETC811 Thermal Cycler (Eastwin Scientific Equipments Inc., Suzhou, China), with the Taq DNA Polymerase Master Mix (KT201, Tiangen Biotech Inc., Beijing, China) and specific primers for the Lep promoter and DMRs of the four altered imprinted genes (*H19*, *Igf2*, *Plagl1*, and *Kcnq1ot1*), which were designed using Methprimer software (The Li Lab, Peking Union Medical College Hospital, Chinese Academy of Medical Sciences) (Supplementary Table 2). The examined promoter regions of Lep and DMRs of the imprinted genes are listed in Supplementary Figure 1. The product DNA sequencing was performed using the method of Sanger sequencing (76) with Applied Biosystems*^rmTM^* ABI3730xl DNA Analyzer (Thermo Fisher Scientific, Waltham, MA, USA), by BGI Tech Solutions (Beijing Liuhe) Co., Ltd. (Beijing, China). Briefly, the single band of the product was confirmed by gel electrophoresis, and polymerase enzymes, unbound primers, and other impurities in the product were eliminated. Then, the sequencing reaction was conducted with the appropriate concentration and ratio between primers and sequencing target. After purification and quantification, the targeted amplicon was loaded on plate using capillary separation for cycle sequencing. The methylation fraction was calculated from the amplitude of cytosine and thymine within each CpG dinucleotide, C/(C + T), as described by Feskens et al. (77).

### 2.7 Statistical analysis

All statistical analyses were conducted using SPSS for Windows (version 21.0, IBM Corp., Armonk, NY, USA). One-way analysis of variance was used to compare the means in different groups when the data were normally distributed. Following analysis of variance, a *post hoc* test, either the Bonferroni or Dunnett’s T3 test, was conducted for data lacking homogeneity of variance. The Kolmogorov–Smirnov test was used to evaluate whether the data were normally distributed, whereas the Kruskal–Wallis test was used for non-normally distributed data. Linear relationships between the variables were tested using Spearman’s correlations. Principal component analysis (PCA) was performed to determine the major variability patterns of the imprinted genes between the different n-3 PUFA diet groups. Statistical significance was set at *P* < 0.05.

## 3 Results

### 3.1 Effects of paternal n-3 PUFAs on body weight and reproduction

As shown in Table 2, fathers fed either the n-3 N or n-3 H diet did not show any changes in their body weight and energy intake. They had increased sperm counts and vitality, with no alterations in litter size and sex ratio in either F1 or F2 offspring. Testis fatty acid analysis showed that the n-3 N and n-3 H diets increased the concentrations of total n-3 PUFAs, EPA, and DHA, with reduced ratios of n-6: n-3 PUFAs and AA:DHA. Furthermore, the effects of the n-3 H diet on testis fatty acids were greater than the n-3 N diet (Table 3).

**TABLE 2:** Effects of paternal n-6: n-3 PUFAs on body weight and reproduction in themselves (F0).

	F0					F1		F2	
	Body weight (g)	Energy intake (kcal/d/mouse)	Sperm counts (×10^6^/L)	Live sperm (×10^6^/L)	Sperm vitality (%)	Litter size	Sex ratio (F/M)	Litter size	Sex ratio (F/M)
n-3 D	24.8 ± 1.9	10.99 ± 0.64	6.70 ± 0.93	4.90 ± 1.19	72.29 ± 9.11	9.6 ± 5.7	0.96	9.0 ± 5.1	1.00
n-3 N	25.1 ± 1.4	10.60 ± 0.44	8.99 ± 0.47*	7.52 ± 0.71*	83.66 ± 7.50*	8.3 ± 5.4	0.98	6.5 ± 5.6	0.77
n-3 H	26.6 ± 1.7	11.04 ± 0.23	10.49 ± 1.82*	9.49 ± 1.88*	90.11 ± 3.85*	9.1 ± 5.2	1.17	10.5 ± 3.6	1.13

Values are means ± *SD*, *n* = 12 in each group for F0. *P < 0.05 as compared to n-3 D group. Statistical differences were analyzed by one-way analysis of variance with Bonferroni *post hoc*. F, female; M, male; n-3 D, n-3 polyunsaturated fatty acid (PUFA)-deficient diet; n-3 N, normal n-3 PUFA content diet; n-3 H, high n-3 PUFA content diet.

**TABLE 3:** Effects of paternal n-6: n-3 PUFAs on testis fatty acid concentrations in themselves (F0).

	n-3 D	n-3 N	n-3 H
Total SFA	42.47 ± 5.76	42.32 ± 6.06	43.48 ± 8.85
Total MUFA	25.90 ± 8.62	27.02 ± 7.12	28.36 ± 8.21
Total n-6 PUFAs	27.86 ± 3.94	25.13 ± 1.69	20.94 ± 1.99*^#^
18:2 n-6 (LA)	9.50 ± 6.50	10.48 ± 3.73	9.71 ± 4.55
18:3 n-6 (GLA)	0.23 ± 0.14	0.30 ± 0.07	0.25 ± 0.19
20:3 n-6 (DGLA)	0.71 ± 0.26	1.04 ± 0.24*	0.95 ± 0.32
20:4 n-6 (AA)	8.83 ± 3.08	7.80 ± 2.17	5.99 ± 2.21
22:2 n-6 (DDA)	0.12 ± 0.09	0.10 ± 0.09	0.07 ± 0.08
22:4 n-6 (ADA)	0.96 ± 0.42	0.64 ± 0.22	0.49 ± 0.20 *
22:5 n-6 (OA)	7.35 ± 2.92	4.74 ± 1.45*	3.48 ± 1.54*^#^
24:4 n-6	0.15 ± 0.14	0.02 ± 0.05*	0.00 ± 0.00*
Total n-3 PUFAs	3.78 ± 2.17	5.53 ± 1.27*	7.19 ± 0.85*^#^
18:3 n-3 (ALA)	0.16 ± 0.06	0.86 ± 0.43*	2.39 ± 1.39*^#^
18:4 n-3 (STA)	0.18 ± 0.07	0.23 ± 0.12	0.21 ± 0.13
20:3 n-3 (EA)	0.89 ± 1.20	0.45 ± 0.16	0.66 ± 0.38
20:5 n-3 (EPA)	0.02 ± 0.05	0.13 ± 0.12*	0.27 ± 0.11*^#^
22:5 n-3 (DPA)	0.07 ± 0.11	0.21 ± 0.24	0.27 ± 0.17*
22:6 n-3 (DHA)	2.06 ± 0.90	3.36 ± 1.11*	3.15 ± 1.07*
24:5 n-3	0.40 ± 0.20	0.29 ± 0.17	0.23 ± 0.21
n-6: n-3 PUFAs	9.30 ± 4.63	4.75 ± 1.08*	2.93 ± 0.27*^#^
AA:DHA	4.46 ± 0.51	2.37 ± 0.19*	1.90 ± 0.19*^#^

Each fatty acid is expressed as the percent (%) (wt/wt) of total fatty acids. Values are means ± *SD*, *n* = 8 in each group. *P < 0.05 as compared to n-3 D group. ^#^*P* < 0.05 as compared to n-3 N group. Statistical differences were analyzed by one-way analysis of variance with Bonferroni *post hoc*. For non-normally distributed data, the Kruskal–Wallis test was used with Mann-Whitney U *post hoc*.

SFA, saturated fatty acids; MUFA, monounsaturated fatty acids; PUFAs, polyunsaturated fatty acids; LA, linoleic acid; GLA, γ-linoleic acid; DGLA, dihomo-γ-linolenic acid; AA, arachidonic acid; DDA, decanedicarboxylic acid; ADA, adrenic acid; OA, osbond acid; ALA, α-linolenic acid; STA, stearidonicacid; EA, eicosatrienoic acid; EPA, eicosapentaenoic acid; DPA, docosapentaenoci acid; DHA, docosahexaenoic acid. n-3 D, n-3 PUFA-deficient diet; n-3 N, normal n-3 PUFA content diet; n-3 H, high n-3 PUFA content diet.

### 3.2 Effects of paternal n-3 PUFAs on offspring Lep expression

**FIGURE 2:**
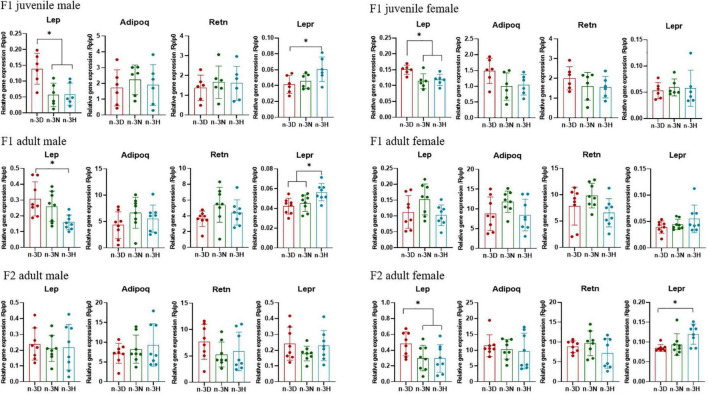
Effects of paternal dietary ratio of n-6: n-3 PUFAs on the expression of Lep, Adipoq, Retn, and Lepr in the adipose tissue of offspring. The protocols for production of F1 and F2 offspring as well as the experiments were the same as those described in Figure 1. Mice from the F1 or F2 offspring were fed normal chow diet for 16 weeks. The mRNA expression of leptin (Lep), adiponectin (Adipoq), and resistin (Retn) in the epididymal fat, as well as, Lep receptor (Lepr) in the hypothalamus, was measured using real-time qPCR. *n* = 6–8 mice in each group. Data have been presented as mean values with standard deviation. *P < 0.05. Statistical differences were analyzed by one-way analysis of variance with Bonferroni *post hoc*, or Dunnett’s T3 test for data lacking homogeneity of variance. For non-normally distributed data, the Kruskal–Wallis test was used with Mann-Whitney U *post hoc*.

As shown in Figure 2, as compared to the n-3 D diet, the paternal n-3 N and n-3 H diets downregulated adipose mRNA expression of Lep in the juvenile F1 males and females, while only adult F1 males (but not females) and adult F2 females (but not males) had downregulated Lep expression in the adipose tissue, with upregulated Lepr expression in the hypothalamus. Consistently, plasma concentrations of leptin in the juvenile F1 offspring (both male and female) and adult F1 males and F2 females were reduced by the paternal n-3 N and n-3 H diets (Table 4). No differences in the adipose expression of Adipoq and Retn were observed among the three paternal diet groups, either in the F1 or F2 offspring (Figure 2). Moreover, the plasma concentrations of total cholesterol, triglycerides, glucose, and insulin were not different among the three paternal diet groups in the offspring (Table 4).

**TABLE 4:** Effects of paternal n-6: n-3 PUFAs on plasma concentrations of leptin and biochemical parameters in the offspring.

	F1 juveniles		F1 adults		F2 adults	
	Male	Female	Male	Female	Male	Female
**Body weight**						
n-3 D	12.8 ± 0.6	12.0 ± 0.8	27.1 ± 1.3	22.5 ± 1.3	25.1 ± 1.0	21.1 ± 0.9
n-3 N	11.5 ± 0.9*	11.4 ± 0.8	26.8 ± 1.6	22.0 ± 0.7	26.1 ± 1.2*	21.1 ± 1.0
n-3 H	11.7 ± 0.6*	11.5 ± 0.7	25.7 ± 0.9*	21.3 ± 1.2	25.5 ± 1.4	21.9 ± 1.5
**Plasma leptin concentration (μ g/L)**						
n-3 D	1.14 ± 0.26	1.57 ± 0.76	1.94 ± 0.66	6.98 ± 1.30	2.28 ± 0.35	9.90 ± 3.51
n-3 N	0.57 ± 0.46*	0.11 ± 0.09*	1.14 ± 0.41*	5.74 ± 2.29	1.90 ± 0.88	3.96 ± 1.43*
n-3 H	0.65 ± 0.32*	0.35 ± 0.25*	1.17 ± 0.19*	5.25 ± 2.74	2.22 ± 1.02	5.29 ± 1.86*
**Plasma triglyceride concentration (mmol/L)**						
n-3 D			1.41 ± 0.21	1.23 ± 0.18	1.31 ± 0.20	1.26 ± 0.20
n-3 N			1.38 ± 0.10	1.04 ± 0.25	1.31 ± 0.05	1.14 ± 0.09
n-3 H			1.59 ± 0.14	1.16 ± 0.28	1.32 ± 0.30	1.25 ± 0.25
**Plasma total cholesterol concentration (mmol/L)**						
n-3 D			4.19 ± 0.46	2.85 ± 0.37	4.60 ± 0.71	3.74 ± 0.42
n-3 N			4.11 ± 0.48	3.19 ± 0.54	4.13 ± 0.75	2.88 ± 0.11*
n-3 H			4.52 ± 0.38	3.26 ± 0.48	4.00 ± 0.94	3.37 ± 0.25
**Plasma glucose concentration (mmol/L)**						
n-3 D			3.49 ± 1.77	5.46 ± 1.42	6.05 ± 1.49	8.15 ± 1.39
n-3 N			4.47 ± 2.16	6.67 ± 2.47	7.16 ± 1.75	9.97 ± 2.63
n-3 H			4.77 ± 0.68	6.95 ± 2.19	6.95 ± 1.50	9.17 ± 1.98
**Plasma insulin concentration (mIU/L)**						
n-3 D			14.95 ± 5.05	17.12 ± 7.98	23.14 ± 7.61	16.89 ± 4.68
n-3 N			13.03 ± 3.56	12.20 ± 4.59	19.30 ± 5.03	18.79 ± 5.35
n-3 H			17.29 ± 8.25	11.11 ± 2.87	16.42 ± 3.41	14.26 ± 2.95

Values are means ± *SD*, *n* = 6–8 in each group. *P < 0.05 as compared to n-3 D group. Statistical differences were analyzed by one-way analysis of variance with Bonferroni *post hoc*. n-3 D, n-3 polyunsaturated fatty acid (PUFA)-deficient diet; n-3 N, normal n-3 PUFA content diet; n-3 H, high n-3 PUFA content diet.

### 3.3 Effects of paternal n-3 PUFAs on the expression of imprinted genes in the adipose tissue of offspring

**FIGURE 3:**
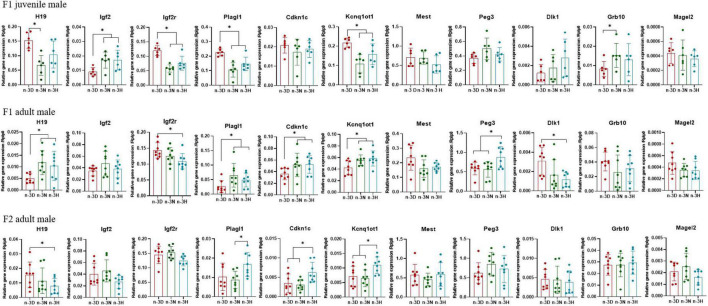
Effects of paternal dietary ratio of n-6: n-3 PUFAs on the expression of imprinted genes in the adipose tissue of male offspring. The protocols for production of F1 and F2 offspring as well as the experiments were the same as those described in Figure 1. Mice from the F1 or F2 offspring were fed normal chow diet for 16 weeks. The mRNA expression of the imprinted genes in the epididymal fat was measured using real-time qPCR. *n* = 6–8 mice in each group. Data have been presented as mean values with standard deviation. *P < 0.05. Statistical differences were analyzed by one-way analysis of variance with Bonferroni *post hoc*, or Dunnett’s T3 test for data lacking homogeneity of variance. For non-normally distributed data, the Kruskal–Wallis test was used with Mann-Whitney U *post hoc*.

**FIGURE 4:**
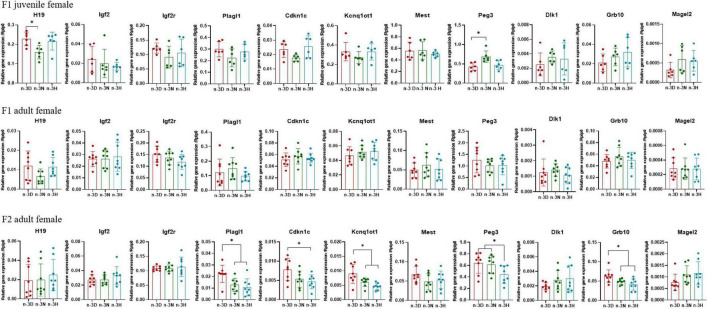
Effects of paternal dietary ratio of n-6: n-3 PUFAs on the expression of imprinted genes in the adipose tissue of female offspring. The protocols for production of F1 and F2 offspring as well as the experiments were the same as those described in Figure 1. Mice from the F1 or F2 offspring were fed normal chow diet for 16 weeks. The mRNA expression of the imprinted genes in the epididymal fat was measured using real-time qPCR. *n* = 6–8 mice in each group. Data have been presented as mean values with standard deviation. *P < 0.05. Statistical differences were analyzed by one-way analysis of variance with Bonferroni *post hoc*, or Dunnett’s T3 test for data lacking homogeneity of variance. For non-normally distributed data, the Kruskal–Wallis test was used with Mann-Whitney U *post hoc*.

Figures 3, 4 depict the effects of paternal n-3 PUFA status on the expression of imprinted genes in the epididymal fat from the F1 and F2 generations. In the F1 offspring, as compared to the n-3 D diet, paternal n-3 N and n-3 H diets downregulated the mRNA expression of *H19*, *Igf2r*, *Plagl1*, and *Kcnq1ot1*, with increased expression of *Igf2* and *Grb10* in juvenile males, but upregulated the mRNA expression of *H19*, *Plagl1*, *Cdkn1c*, *Kcnq1ot1*, and *Peg3*, with decreased expression of *Igf2r* and *Dlk1* in adult males. Little or no effect of paternal n-3 N or n-3 H diet was observed on the expression of these genes in either juvenile or adult females. In case of the adult F2 offspring, only the paternal n-3 H diet altered the expression of imprinted genes, with downregulation of *H19* and upregulation of *Cdkn1c* and *Kcnq1ot1* in males; whereas, either paternal n-3 N or n-3 H diet downregulated the expression of *Plagl1*, *Cdkn1c*, *Kcnq1ot1*, *Peg3*, and *Grb10* in females, indicating that female offspring are more easily affected by paternal n-3 PUFAs than male offspring.

### 3.4 Correlation between leptin and imprinted genes in the adipose tissue

**FIGURE 5:**
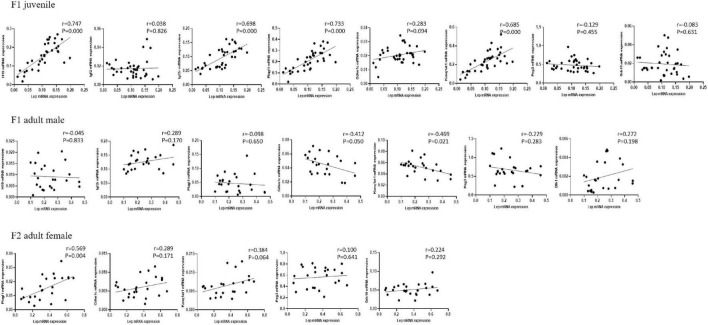
Correlation between the expression of Lep and imprinted genes in the adipose tissue. The protocols for production of F1 and F2 offspring as well as the experiments were the same as those described in Figure 1. Mice from the F1 and F2 offspring were fed normal chow diet for 16 weeks. Linear correlation analysis was performed to compare the expression of leptin (Lep) and other altered imprinted genes in juvenile F1 males and females, adult F1 males, and adult F2 females.

Linear correlation analysis indicated that adipose leptin expression was positively correlated with the expression of *H19*, *Igf2r*, *Plagl1*, and *Kcnq1ot1* in juvenile F1 males and females; negatively correlated with the expression of *Kcnq1ot1* in adult F1 males, with a negative trend toward significance for the *Cdkn1c* expression; and positively correlated with the expression of *Plagl1* in adult F2 females, with a positive trend toward significance for the *Kcnq1ot1* expression (Figure 5). PCA identified 3–4 components with an eigenvalue ≥ 1, which accounted for 65.831, 73.031, and 65.351% of the total variance in juvenile F1 males and females, adult F1 and F2 males, and adult F1 and F2 females, respectively, with the first factor accounting for 34.702, 35.629, and 41.561% of the variance, respectively (Table 5). Supplementary Table 3 shows the variables that accounted for the majority of variance in the data. The first factor was predominantly loaded by *H19*, *Igf2r*, *Plagl1*, *Cdkn1c*, and *Kcnq1ot1* in juvenile F1 males and females; *Plagl1*, *Cdkn1c*, *Kcnq1ot1*, *Mest*, *Dlk1*, and *Magel2* in all adult F1 and F2 males; and *Igf2r*, *Plagl1*, *Cdkn1c*, *Kcnq1ot1*, *Peg3*, *Dlk1*, and *Magel2* in adult F1 and F2 females.

**TABLE 5:** The total variance explained for the offspring dataset.

	Component	Eigenvalue	Variance (%)	Cumulative (%)
F1 juveniles	1	3.817	34.702	34.702
	2	1.726	15.690	50.392
	3	1.698	15.439	65.831
Adult males	1	3.919	35.629	35.629
	2	1.544	14.034	49.663
	3	1.380	12.546	62.209
	4	1.190	10.822	73.031
Adult females	1	4.572	41.561	41.561
	2	1.355	12.317	53.878
	3	1.262	11.473	65.351

### 3.5 Effects of paternal n-3 PUFAs on the expression of imprinted genes in the offspring testis

**FIGURE 6:**
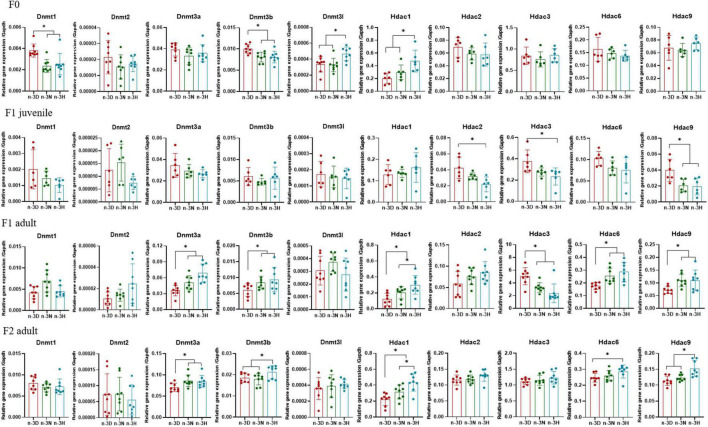
Effects of paternal dietary ratio of n-6: n-3 PUFAs on the expression of Dnmts and Hdacs in the offspring testis. The protocols for production of F1 and F2 offspring as well as the experiments were the same as those described in Figure 1. Mice from the F1 or F2 offspring were fed normal chow diet for 16 weeks. The mRNA expression of DNA methyltransferase (Dnmts) and histone deacetylases (Hdacs) in the testis was measured using real-time qPCR. *n* = 6–8 mice in each group. Data have been presented as mean values with standard deviation. *P < 0.05. Statistical differences were analyzed by one-way analysis of variance with Bonferroni *post hoc*, or Dunnett’s T3 test for data lacking homogeneity of variance. For non-normally distributed data, the Kruskal–Wallis test was used with Mann-Whitney U *post hoc*.

**FIGURE 7:**
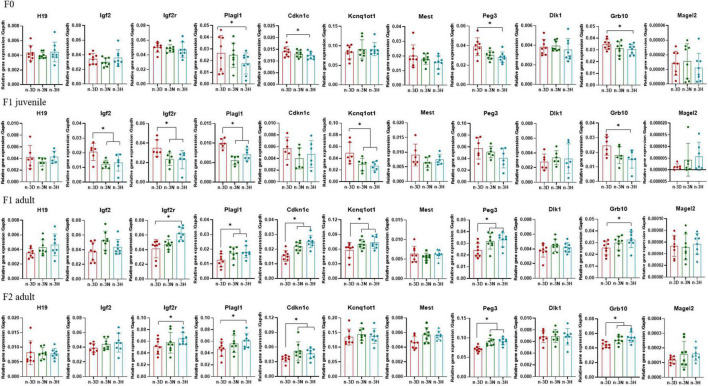
Effects of paternal dietary ratio of n-6: n-3 PUFAs on the expression of imprinted genes in the offspring testis. The protocols for production of F1 and F2 offspring as well as the experiments were the same as those described in Figure 1. Mice from the F1 or F2 offspring were fed normal chow diet for 16 weeks. The mRNA expression of the imprinted genes in the testis was measured using real-time qPCR. *n* = 6–8 mice in each group. Data have been presented as mean values with standard deviation. *P < 0.05. Statistical differences were analyzed by one-way analysis of variance with Bonferroni *post hoc*. For non-normally distributed data, the Kruskal–Wallis test was used with Mann-Whitney U *post hoc*.

Figures 6, 7 depict the effects of paternal n-3 PUFA status on the expression of imprinted genes in the testes from the F1 and F2 generations. Paternal n-3 H diet downregulated the mRNA expression of *Plagl1*, *Cdkn1c*, *Peg3*, and *Grb10*, with altered expression of Dnmts (Dnmt1, Dnmt3b, and Dnmt3l) and Hdac1 (founder F0 fathers). Examination of the imprinted genes in F1 testes revealed that the paternal n-3 N and n-3 H diets downregulated the expression of *Igf2*, *Igf2r*, *Plagl1*, *Kcnq1ot1*, and *Grb10*, and decreased the expression of Hdac2, Hdac3, and Hdac9 in juvenile offspring, but upregulated the expression of *Igf2r*, *Plagl1*, *Cdkn1c*, *Kcnq1ot1*, *Peg3*, and *Grb10*, with increased expression of Dnmt3a, Dnmt3b, Hdac1, Hdac6, and Hdac9 in adult offspring. Similar to the observations in F1 adults, the paternal n-3 N and n-3 H diets upregulated the expression of *Igf2r*, *Plagl1*, *Cdkn1c*, *Peg3*, and *Grb10*, with increased expression of Dnmt3a, Dnmt3b, Hdac1, Hdac6, and Hdac9 in the testes of adult F2 offspring.

### 3.6 Effects of paternal n-3 PUFAs on gene DNA methylation in the offspring

Alterations in the average DNA methylation fractions of the leptin promoter in the adipose tissue was only observed upon administration of the paternal n-3 H diet, and in adult F1 males. Paternal n-3 N and n-3 H diets decreased the average DNA methylation fractions of *H19* in adult F1 males, and *Igf2* DMRs in both juvenile and adult F1 males, but elevated those of *Igf2* DMR in adult F2 males. Meanwhile, paternal n-3 N and n-3 H diets increased the average DNA methylation fractions of *Kcnq1ot1* DMR in adult F1 males, but decreased them in adult F2 males and females (Table 6). The methylation fractions for the specific CpG sites are shown in Supplementary Tables 4–8.

**TABLE 6:** Effects of paternal n-6: n-3 PUFAs on DNA methylation of the leptin promoter and DMRs of imprinted genes in the adipose tissue.

	F1 juvenile		F1 adult		F2 adult	
	Male	Female	Male	Female	Male	Female
**Leptin promoter**						
n-3 D	0.79 ± 0.03	0.80 ± 0.02	0.73 ± 0.04	073 ± 0.02	0.78 ± 0.05	0.74 ± 0.03
n-3 N	0.81 ± 0.01	0.78 ± 0.02	0.72 ± 0.04	0.72 ± 0.02	0.78 ± 0.05	0.75 ± 0.04
n-3 H	0.81 ± 0.02	0.80 ± 0.02	0.70 ± 0.04*	0.72 ± 0.02	0.74 ± 0.03	0.74 ± 0.03
**H19 DMR**						
n-3 D	0.73 ± 0.01	0.72 ± 0.02	0.81 ± 0.02	0.75 ± 0.01	0.70 ± 0.01	0.70 ± 0.03
n-3 N	0.70 ± 0.01	0.74 ± 0.01	0.77 ± 0.01*	0.76 ± 0.01	0.70 ± 0.02	0.69 ± 0.02
n-3 H	0.71 ± 0.03	0.73 ± 0.02	0.76 ± 0.01*	0.75 ± 0.02	0.69 ± 0.02	0.70 ± 0.01
**Igf2 DMR**						
n-3 D	0.69 ± 0.03	0.67 ± 0.03	0.68 ± 0.01	0.66 ± 0.03	0.66 ± 0.03	0.65 ± 0.01
n-3 N	0.69 ± 0.01	0.66 ± 0.02	0.67 ± 0.02	0.66 ± 0.01	0.65 ± 0.02	0.64 ± 0.01
n-3 H	0.64 ± 0.01*^#^	0.67 ± 0.02	0.66 ± 0.02*	0.64 ± 0.01	0.70 ± 0.03*^#^	0.65 ± 0.01
**Plagl1 DMR**						
n-3 D	0.69 ± 0.02	0.67 ± 0.01	0.68 ± 0.02	0.67 ± 0.02	0.71 ± 0.01	0.71 ± 0.02
n-3 N	0.68 ± 0.02	0.67 ± 0.03	0.69 ± 0.02	0.68 ± 0.01	0.70 ± 0.02	0.72 ± 0.02
n-3 H	0.69 ± 0.01	0.66 ± 0.01	0.69 ± 0.03	0.65 ± 0.02*^#^	0.70 ± 0.01	0.71 ± 0.01
**Kcnq1ot1 DMR**						
n-3 D	0.69 ± 0.01	0.70 ± 0.01	0.70 ± 0.03	0.68 ± 0.01	0.75 ± 0.02	0.68 ± 0.03
n-3 N	0.68 ± 0.01	0.69 ± 0.02	0.70 ± 0.02	0.69 ± 0.01	0.67 ± 0.03*	0.66 ± 0.02
n-3 H	0.68 ± 0.01	0.68 ± 0.01	0.74 ± 0.03*^#^	0.70 ± 0.03	0.69 ± 0.01*	0.65 ± 0.01*

Data are averaged methylation fractions, which are calculated from the amplitude of cytosine and thymine within each CpG dinucleotide C/(C + T). The result for each CpG site is represented as the mean values and standard deviations determined from six to eight mice in each group. *P < 0.05 as compared to n-3 D group. ^#^*P* < 0.05 as compared to n-3 N group.

Statistical differences were analyzed by one-way analysis of variance with Bonferroni *post hoc*.

DMR, differentially methylated region; n-3 D, n-3 polyunsaturated fatty acid (PUFA)-deficient diet; n-3 N, normal n-3 PUFA content diet; n-3 H, high n-3 PUFA content diet.

## 4 Discussion

In the current study, paternal pre-conceptional supplementation with n-3 PUFAs reduced adipose expression and plasma concentrations of leptin in juvenile F1 males and females, as well as adult F1 males and F2 females, accompanied by upregulated expression of Lepr in the hypothalamus. Meanwhile, the expression of imprinted genes (*H19*, *Igf2*, *Igf2r*, *Plagl1*, *Cdkn1c*, *Kcnq1ot1*, *Peg3*, and *Grb10*) in the adipose tissue was greatly altered in juvenile or adult F1 males, but not F1 females, upon paternal n-3 PUFA supplementation. However, in adult F2 offspring, greater effects of paternal n-3 PUFAs on imprinted gene expression were observed in females than in males. In addition, the expression of imprinted genes (*Igf2*, *Igf2r*, *Plagl1*, *Cdkn1c*, *Kcnq1ot1*, *Peg3*, or *Grb10*) in the testes was altered by paternal n-3 PUFAs, with altered expression of epigenetic regulators, Dnmt3 (3a and 3b) and Hdacs (1, 2, 3, 6, and 9), in juvenile or adult F1 offspring, as well as adult F2 offspring.

In keeping with the reduced effects of paternal n-3 PUFA supplementation on offspring Lep expression described herein, studies on maternal intervention have shown that maternal n-3 PUFA supplementation lowers leptin levels in the offspring (31–33). The lower leptin levels caused by parental n-3 PUFA supplementation might be helpful for the offspring’s health, because relatively lower leptin levels during early life or adulthood can improve the development of central and peripheral systems (30–34, 36) and further prevent the occurrence of metabolic diseases in later life (24–28). Adipoq and Retn, which are adipokines secreted by adipose tissue, are important for maintaining homeostasis of insulin action, energy, glucose, lipids, and inflammation (78, 79). In our study, paternal n-3 PUFA supplementation had no effect on the adipose expression of Adipoq and Retn, in both the F1 and F2 generations. To date, only a few studies have demonstrated that maternal DHA supplementation during pregnancy and lactation increases adipose expression of Adipoq in animals, and that higher maternal n-3 PUFAs are inversely related to Adipoq expression in early childhood; however, none of these associations were found to persist in mid-childhood (80, 81).

Interestingly, we found that paternal n-3 PUFAs reduced leptin levels in adult offspring in a sex-specific manner, with effects on F1 males and F2 females. In keeping with this, peri-conceptional exposure to other nutritional changes (e.g., obesity, fructose-rich diet, and Zn deficiency) is sufficient to shape offspring expression patterns of metabolism-associated genes, characterized by impacts on males instead of females in the F1 generation (82–86). However, inconsistent results have also been reported, with female (but not male) offspring from fathers fed a high-fat diet before mating displaying impaired pancreatic β-cell function with increased body weight (87). In addition, maternal and paternal deficiency of endothelial nitric oxide synthase, which mediates the effects of n-3 PUFAs on leptin expression in endothelial progenitor cells (88), determines the mouse fatty liver phenotype in female and male offspring, respectively (89, 90). A study on F2 offspring indicated that paternal pre-conceptional high- fat-, sucrose-, and salt-rich diets predispose female F2 offspring to chronic kidney disease in rats (91). Herein, the variations in leptin expression between the sexes might also be due to biological differences. It has been demonstrated that sexual dimorphism in leptin plasma levels and adipose mRNA expression is apparent early in life and becomes more marked with age, and this can be influenced by food intake, diurnal rhythm, and species (92–95). Male mice in the fed state had higher (92) or similar (93) circulating leptin levels than female mice, probably because of the increased adipose tissue mass apparent in males, as compared to females (92, 94). However, during fasting, the reduction in leptin was more pronounced in males than in females, resulting in a higher plasma leptin level after fasting in females (93), which is consistent with the findings in the fasted state in the current study. In humans, females naturally have higher fat mass and circulating serum leptin concentrations than males, which may mask any association between paternal n-3 PUFAs and leptin expression (95). The same was true for the mouse model used in the present study.

It has been reported that paternal nutrition before conception (such as a high-fat diet) can over multiple generations have effects on offspring metabolic traits, including the expression of Adipoq and Lep, and their gene promoter methylation and histone modification in the adipose tissue (96). In this study, paternal n-3 H diet caused a reduction in the average DNA methylation fractions of the Lep promoter exclusively in adult male F1 offspring, which could not explain for the downregulated Lep mRNA expression caused by both paternal n-3 N and n-3 H diets in both juvenile and adult F1 males as well as adult F2 females. Thus, whether other types of epigenetic pathways, such as histone modification and altered binding domain proteins (36) at the Lep promoter, might contribute to the effects of paternal n-3 PUFAs on offspring leptin programming needs to be elucidated in the future.

Furthermore, determination of the imprinted genes associated with growth and development showed that the effects of paternal n-3 N and n-3 H diets on gene imprinting in the adipose tissue of juvenile and adult F1 males and adult F2 females were similar, with altered expression of *H19*, *Plagl1*, *Cdkn1c*, *Kcnq1ot1*, *Peg3*, or *Grb10* being common. PCA results showed that besides *H19* and *Igf2r* in juveniles, and *Mest*, *Dlk1*, and *Magel2* in adults, *Plagl1*, *Cdkn1c*, and *Kcnq1ot1* contributed the most to variation in adipose tissue expression in all offspring. Linear correlation analysis indicated that most of these genes were closely correlated with Lep mRNA expression, implying that they may be involved in the inhibitory effects of paternal n-3 PUFA supplementation on leptin production. This is consistent with other reports that imprinted genes may regulate leptin expression (47–51, 61–68). Simultaneously, the testes in juvenile F1 offspring, and adult F1 and F2 offspring had altered expression of the same genes, *Plagl1*, *Cdkn1c*, *Kcnq1ot1*, *Peg3*, and *Grb10*, upon paternal n-3 PUFA supplementation. This hints at the possibility that the paternal n-3 PUFA supplementation-induced epigenetic memory, particularly imprinting of genes (*Plagl1*, *Cdkn1c*, and *Kcnq1ot1*), which occurs during germ cell development, might contribute to transgenerational transmission.

Moreover, the paternal n-3 PUFA-mediated alterations in adipose expression of the above-mentioned imprinted genes occurred in opposite directions between juvenile and adult male F1 offspring, and between adult male F1 offspring and female F2 offspring. However, in these offspring mice, leptin expression in the adipose tissue was consistently downregulated by paternal n-3 PUFA supplementation. Therefore, the positive or negative effects of imprinted genes on leptin production and related metabolic processes may be complicated. Studies have indicated that spatiotemporal regulation of imprinted genes and their activities vary with life stages, sex, and genetic background (47, 97, 98). For example, Bergmann et al. found that deletion of *Nnat* in mice of the 129S2/Sv background, but not the C57BL/6J background, causes postnatal growth-restriction with reduced adipose tissue accumulation, followed by catch-up growth after weaning (98). Male mice that inherit a *Peg3* deletion from their father have growth retardation at birth and delayed adipose accumulation after weaning, but enhanced white adipose deposition with high levels of circulating leptin in adulthood (47). However, females with an ablated *Peg3* gene are poor mothers who fail to increase food intake in early gestation and consequently have a reduced postpartum adipose reserve (97). Thus, in our study, the paternal n-3 PUFA supplementation-mediated increased expression of *Peg3* observed in F1 males, but decreased expression observed in F2 females, may explain the decrease in leptin expression in both adult F1 males and F2 females. However, it remains unclear whether this phenomenon can be extended to other imprinted genes, among which the expression of *Plagl1*, *Cdkn1c*, *Kcnq1ot1*, and *Peg3*, was upregulated in adult F1 males, but downregulated in adult F2 females, upon paternal n-3 PUFA supplementation.

The parent-of-origin-specific acquisition of DNA methylation marks at DMRs in the germline is essential for the monoallelic expression of imprinted genes in embryos, and is retained as the memory of parental origin after fertilization (99). Generally, methylation of imprinting control region 1 (ICR1) at the *H19*/*IGF2* locus and unmethylation of ICR2 at the *Cdkn1c*/*Kcnq1ot1* locus on the paternal allele initiates the expression of *Igf2* and *Kcnq1ot1*, with silenced expression of *H19* and *Cdkn1c* (46). In the present study, we found that the paternal n-3 H diet decreased the average DNA methylation fractions of *H19* DMR (ICR1) and *Igf2* DMR in both juvenile and adult F1 males, but elevated them in adult F2 males. Meanwhile, the paternal n-3 N and n-3 H diets increased the average DNA methylation fractions of *Kcnq1ot1* DMR (ICR2) in adult F1 males, but decreased them in adult F2 males and females. Alterations in DMR methylation of these imprinted genes did not match their mRNA expression levels. This might be attributable to the contribution of other pathways, such as histone modification and non-coding RNA (6), which need to be investigated in the future.

Accumulating evidence indicates that the n-6: n-3 PUFAs balance is an important factor in the maintenance of body homeostasis, normal development, and mental health throughout the life cycle, with a n-6: n-3 PUFAs ratio of 1:1–2:1 being considered an optimal target for health (71). Maternal intervention studies have demonstrated that reduction of the n-6: n-3 PUFA ratio from the range of 9–7:1 to that of 3.5–1:1 has more beneficial effects on problem-solving in infancy and obesity-prevention at preschool age (100–102). In the current study, we found that a paternal diet with an n-6: n-3 ratio of 1.5:1 altered more parameters than one with a ratio of 4.3:1, in both the adipose tissue and testis of mice, thereby implying a dose-dependent effect of n-3 PUFAs, to some extent.

To our knowledge, this is the first study to confirm the effects of the paternal dietary ratio of n-6: n-3 PUFAs on leptin expression and associated gene imprinting in offspring. However, some limitations of the current study must be addressed. Although several imprinted genes have been shown to influence leptin expression, the possibility that alterations in leptin expression result from adiposity changes induced by these imprinted genes cannot be ruled out. It is not known whether the imprinted gene products directly regulate leptin expression, neither is the underlying mechanism(s); this question has not been answered in the current study as well. Thus, the causal relationship between imprinted genes and leptin expression should be investigated intensively, as it may clarify the role of diet- and environment-associated gene imprinting in health and disease.

In summary, in mice, a lower paternal n-6: n-3 PUFA ratio with n-3 PUFA supplementation during pre-conception reduced the adipose mRNA expression of Lep and its plasma levels in juvenile male and female F1 offspring as well as adult male F1 and female F2 offspring, with concomitant alterations in the expression of imprinted genes, both in the adipose tissue and testis. These data suggest that paternal n-3 PUFAs program leptin expression, possibly through gene imprinting in a sex-specific manner over successive generations, and thus, may be beneficial for offspring growth and long-term health. It would be of extreme value to verify the findings of this study in humans.

## Data availability statement

The original contributions presented in this study are included in the article/Supplementary material, further inquiries can be directed to the corresponding author.

## Ethics statement

The animal study was reviewed and approved by the Ethic Committee of the National Institute of Occupational Health and Poison Control, China CDC.

## Author contributions

QS and KQ contributed to the study conception and design and wrote the manuscript. QS and XL performed the experiments and collected the data. XF and RW performed the statistical analyses. All authors commented on the previous versions of the manuscript and approved the final manuscript.

## Footnotes

1
http://www.ncbi.nlm.nih.gov/tools/primer-blast/
