# Supplementary material for: Paternal dietary ratio of n-6: n-3 polyunsaturated fatty acids programs offspring leptin expression and gene imprinting in mice
Source: Front Nutr. 2022 Dec 23;9:1043876. doi: 10.3389/fnut.2022.1043876 (PMC9816484; doi:10.3389/fnut.2022.1043876)
Supplement: Supplementary file 1 [file Data_Sheet_1.docx]

Supplementary Material

**Table 1**. **Sequences of primers used for RT-PCR**

| **primer** |  | **sequence** |
| --- | --- | --- |
| Lep | forward | TGGCTTTGGTCCTATCTGTC |
|  | reverse | TCCTGGTGACAATGGTCTTG |
| Lepr | forward | TGGTCCCAGCAGCTATGGT |
|  | reverse | ACCCAGAGAAGTTAGCACTGT |
| Adipoq | forward | TGACGACACCAAAAGGGCTC |
|  | reverse | ACCTGCACAAGTTCCCTTGG |
| Retn | forward | CCTGCTAAGTCCTCTGCCAC |
|  | reverse | GGCTTCATCGATGGGACACA |
| H19 | forward | GCACTAAGTCGATTGCACTGG |
|  | reverse | CCTCAAGCACACGGCCACACC |
| Igf2 | forward | CGCTTCAGTTTGTCTGTTCG |
|  | reverse | AAGCAGCACTCTTCCACGAT |
| Igf2r | forward | CCACACTGATTACCTTCCTCTGT |
|  | reverse | TACCACCGGAAGTTGTAGGTAGA |
| Plagl1 | forward | GGAAAACAGGAATGGGGTTT |
|  | reverse | TCCCCTTCCTAGGCTACACA |
| Cdkn1c | forward | GCGCAAACGTCTGAGATGAGT |
|  | reverse | AGAGTTCTTCCATCGTCCGCT |
| Kcnq1ot1 | forward | GCACTCTGGGTCCTGTTCTC |
|  | reverse | CACTTCCCTGCCTCCTACAC |
| Peg3 | forward | TCAATGACCTCACAAGCCACCAG |
|  | reverse | CGGGCAACAGAGCGATGAAAGC |
| Dlk1 | forward | ACGGGAAATTCTGCGAAATAG |
|  | reverse | AGCATTCGTACTGGCCTTTCT |
| Grb10 | forward | AGGATCATCAAGCAACAAGGTCTC |
|  | reverse | ATTACTCTGGCTGTCACGAAGGA |
| Mest | forward | CAACAATGACGGCAACCTGGT |
|  | reverse | TCTGAATTTCTTCCTTTGATTAATGTACTGTA |
| Magel2 | forward | ATGGCTCCATCAGGAGAAC |
|  | reverse | GATGGAAAGACCCTTGAGGT |
| Dnmt1 | forward | GCTACCAGTGCACCTTTGGT |
|  | reverse | ATGATGGCCCTCCTTCGT |
| Dnmt2 | forward | AGAAAGGGACAGGAAACA |
|  | reverse | CAATAACTTGGGTGGTAAA |
| Dnmt3a | forward | ACACAGGGCCCGTTACTTCT |
|  | reverse | TCACAGTGGATGCCAAAGG |
| Dnmt3b | forward | GCCTGCAAGACTTCTTCACTACT |
|  | reverse | GGTACAACTTGGGTGGCTCA |
| Dnmt3l | forward | AACCGACGGAGCATTGAA |
|  | reverse | CCGAGTGTACACCTGGAGAGT |
| Hdac1 | forward | CTTACGAAACAGCGGTG |
|  | reverse | CTTCTCCAGGTACTCGTTA |
| Hdac2 | forward | TGCTGAAGAAATGACTAAATACC |
|  | reverse | CAAAGAGTCCATCAAACAC |
| Hdac3 | forward | AATGTGCCCTTACGAGATGG |
|  | reverse | GTAGCCACCACCTCCCAGTA |
| Hdac6 | forward | GGGTTCTCAGCACTTTGGAG |
|  | reverse | TCCTTGTGTCAGCATCAAGC |
| Hdac9 | forward | GCGGTCCAGGTTAAAACAGA |
|  | reverse | GACCTGACCCTGGAGAGCTG |
| Gapdh | forward | CAATAATGGGGAGAGGTTCG |
|  | reverse | TGCTGCTTCCCGAGTAAAAT |
| Rplp0 | forward | AGATTCGGGATATGCTGTTGGC |
|  | reverse | TCGGGTCCTAGACCAGTGTTC |

Abbreviations: Lep, leptin; Lepr, leptin receptor; Adipoq, adiponectin; Retn, resistin; H9, histocompatibility 9; Igf2, insulin-like growth factor 2; Igf2r, insulin-like growth factor 2 receptor; Plag1, pleiomorphic adenoma gene 1; Cdkn1c, cyclin-dependent kinase inhibitor 1C; Kcnq1ot1, KCNQ1 overlapping transcript 1; Peg3, paternally expressed 3; Dlk1, delta like non-canonical Notch ligand 1; Grb10, growth factor receptor bound protein 10; Mest, mesoderm specific transcript; Magel2, MAGE family member L2; Dnmt, DNA methyltransferase; Hdac, histone deacetylases. Gapdh, glyceraldehyde-3-phosphate dehydrogenase; Rplp0, Ribosomal protein large P0.

**Table 2. Bisulfite sequencing primers and annealing temperature in this study**

| **gene** | **region** | **sequences 5‘-3’** | **Product (bp)** | | **Annealing**  **temperature (℃)** |
| --- | --- | --- | --- | --- | --- |
| Lep | Promoter | F out:5'-GAGTAGTTAGGTTAGGTATGTAAAGAG | | 310 | 51 |
|  |  | F in:5'-AGTTTTTTGTAGTTTTTTGTTTTTTG | |  |  |
|  |  | R out:5'-TAATAACTACCCCAATACCACTTAC | |  |  |
| H19 | DMR-1 | F out:5'-AAAAAGGTTGGTGAGAAAATAGAG | | 288 | 55 |
|  |  | R in:5'-AAATAACCCACAACATTACCATTT | |  |  |
|  |  | R out:5'-ACATTTATAACCCCCCTAAAATAA | |  |  |
|  | DMR-2 | F out:5'-GTATTTTAGGGGGGTTATAAATGT | | 220 | 58 |
|  |  | F in:5'-GTAGGATATATGTATTTTTTAGGTTGG | |  |  |
|  |  | R out:5'-CCTCATAAAACCCATAACTATAAAATC | |  |  |
| Igf2 | DMR2 | F out:5'-AGATGGGAGTTTAGGTTAATATGATA | | 285 | 56.5 |
|  |  | R in:5'- CCACATAATTTAATTCACTAATAATTAC | |  |  |
|  |  | R out:5'- TCACAAATTAATAATACTACATTACAA | |  |  |
| Kcnq1ot1 | DMR | F out:5'-TGAGTTTTAAGGTGAGTGGT | | 271 | 59 |
|  |  | F in:5'-TAAGGTGAGTGGTTTAGGAT | |  |  |
|  |  | R out:5'-AATAAACAACCTTCCCCAAC | |  |  |
| Plagl1 | DMR | Fout:5'-GGGTAGGTAAGTAGTGATAA | | 383 | 58 |
|  |  | F in:5'-ATTTGGGTGTTTTAGTTGTA | |  |  |
|  |  | R in:5'-TACAAAACCAAAACCCTTAC | |  |  |
|  |  | R out:5'-CCTAAAACACCAAAATAACA | |  |  |

DMR, differentially methylated regions.

**Table 3.** **Rotated factors with variables loadings against each component accounting for the majority of the variance in the data**

|  | F1 juveniles | | | Adult males | | | | Adult females | | |
| --- | --- | --- | --- | --- | --- | --- | --- | --- | --- | --- |
| Factor | 1 | 2 | 3 | 1 | 2 | 3 | 4 | 1 | 2 | 3 |
| H19 | **0.945** |  |  | 0.132 | **-0.817** |  |  | -0.480 | 0.187 | 0.311 |
| Igf2 | 0.156 | **0.684** | -0.271 |  |  |  | **0.938** |  |  | **0.860** |
| Igf2r | **0.836** | -0.198 | -0.225 | 0.287 |  | **-0.684** | 0.395 | **0.536** | **-0.517** | 0.285 |
| Plagl1 | **0.935** |  |  | -**0.753** |  |  |  | **0.797** |  | 0.116 |
| Cdkn1c | **0.614** | 0.225 |  | **-0.898** |  | 0.108 | 0.140 | **0.933** |  |  |
| Kcnq1ot1 | **0.874** | 0.157 |  | -**0.910** | 0.126 | 0.156 | 0.145 | **0.941** |  |  |
| Mest | -0.219 | -0.138 | -0.458 | **0.771** |  |  | 0.133 |  | **0.560** | 0.402 |
| Peg3 | -0.169 | **0.712** | 0.203 | 0.191 | 0.224 | **0.804** | 0.278 | **0.669** | 0.198 | -0.340 |
| Dlk1 | -0.182 | 0.294 | **0.766** | **0.550** | 0.270 | -0.435 |  | **-0.681** |  | 0.188 |
| Grb10 | 0.254 | **0.690** | 0.392 |  | **0.768** | 0.168 |  |  | **0.824** | -0.110 |
| Magel2 | -0.102 | -0.227 | **0.750** | **0.825** | -0.363 |  |  | **-0.860** |  |  |

Factors with loadings ≥±0.50 were considered significant.

**Table 4. Quantitative methylation analysis of the Lep promoter**

|  | |  | CG1 | CG2 | CG3 | CG4 | CG5 | CG6 | CG7 | CG8 | CG9 | CG10 | CG11 | CG12 | CG13 | CG14 | CG15 | CG16 |
| --- | --- | --- | --- | --- | --- | --- | --- | --- | --- | --- | --- | --- | --- | --- | --- | --- | --- | --- |
| F1 juvenile | | |  |  |  |  |  |  |  |  |  |  |  |  |  |  |  |  |
| Male | n-3D | | 0.66±0.07 | 0.76±0.06 | 0.67±0.06 | 0.74±0.07 | 0.77±0.04 | 0.71±0.06 | 0.80±0.04 | 0.81±0.07 | 0.71±0.06 | 0.79±0.06 | 0.78±0.06 | 0.86±0.03 | 0.86±0.02 | 0.85±0.02 | 0.89±0.01 | 0.92±0.01 |
|  | n-3N | | 0.66±0.03 | **0.83±0.03*** | 0.74±0.04 | 0.76±0.03 | **0.82±0.02*** | 0.73±0.04 | 0.81±0.01 | 0.83±0.02 | 0.71±0.03 | 0.79±0.02 | 0.79±0.02 | 0.86±0.01 | 0.88±0.01 | 0.86±0.02 | 0.90±0.01 | **0.94±0.01*** |
|  | n-3H | | 0.70±0.04 | 0.80±0.04 | **0.75±0.05*** | 0.75±0.04 | 0.81±0.03 | 0.75±0.04 | 0.83±0.02 | 0.84±0.03 | 0.71±0.05 | 0.80±0.04 | 0.79±0.05 | 0.87±0.03 | 0.87±0.04 | 0.87±0.02 | 0.88±0.03 | 0.92±0.04 |
| Female | n-3D | | 0.68±0.05 | 0.80±0.05 | 0.65±0.04 | 0.74±0.05 | 0.79±0.03 | 0.71±0.03 | 0.81±0.03 | 0.84±0.03 | 0.68±0.03 | 0.79±0.02 | 0.79±0.03 | 0.87±0.02 | 0.88±0.01 | 0.86±0.01 | 090±0.01 | 0.94±0.01 |
|  | n-3N | | 0.65±0.03 | 0.78±0.02 | 0.64±0.03 | 0.73±0.02 | 0.79±0.03 | 0.70±0.04 | 0.79±0.03 | 0.82±0.02 | 0.68±0.03 | 0.77±0.02 | 0.78±0.03 | 0.86±0.02 | 0.86±0.03 | **0.84±0.02*** | 0.88±0.03 | 0.93±0.01 |
|  | n-3H | | 0.70±0.05 | 0.81±0.03 | 0.66±0.02 | 0.76±0.03 | 0.82±0.02 | 0.72±0.03 | 0.81±0.02 | 0.85±0.01 | 0.71±0.02 | 0.80±0.02 | 0.79±0.02 | 0.86±0.03 | 0.86±0.04 | 0.85±0.02 | 0.88±0.02 | 0.92±0.02 |
| F1 adult | | |  |  |  |  |  |  |  |  |  |  |  |  |  |  |  |  |
| Male | n-3D | | 0.61±0.03 | 0.56±0.05 | 0.70±0.04 | 0.57±0.03 | 0.65±0.04 | 0.74±0.04 | 0.67±0.03 | 0.76±0.03 | 0.80±0.02 | 0.68±0.03 | 0.77±0.03 | 0.78±0.02 | 0.85±0.02 | 0.86±0.01 | 0.83±0.02 | 0.84±0.02 |
|  | n-3N | | 0.60±0.02 | 0.56±0.03 | 0.68±0.04 | 0.56±0.02 | 0.64±0.04 | 0.74±0.03 | 0.66±0.02 | 0.75±0.03 | 0.78±0.03 | 0.66±0.03 | 0.77±0.02 | 0.78±0.01 | 0.85±0.01 | 0.86±0.01 | 0.84±0.01 | 0.84±0.02 |
|  | n-3H | | 0.58±0.06 | 0.51±0.07 | 0.64±0.08 | 0.51±0.07 | 0.60±0.07 | 0.70±0.05 | **0.62±0.03*** | **0.71±0.04*** | **0.73±0.03*** | **0.61±0.03*** | **0.73±0.04*** | 0.77±0.03 | 0.85±0.02 | 0.87±0.01 | 0.84±0.02 | **0.87±0.01*#** |
| Female | n-3D | | 0.59±0.06 | 0.58±0.05 | 0.65±0.02 | 0.54±0.05 | 0.67±0.06 | 0.79±0.03 | 0.66±0.02 | 0.69±0.06 | 0.71±0.06 | 0.65±0.05 | 0.80±0.03 | 0.80±0.04 | 0.87±0.02 | 0.86±0.03 | 0.86±0.02 | 0.88±0.02 |
|  | n-3N | | 0.59±0.05 | 0.55±0.03 | 0.64±0.03 | 0.52±0.05 | 0.62±0.04 | **0.72±0.04*** | 0.63±0.04 | **0.75±0.03*** | **0.77±0.04*** | 0.66±0.03 | **0.76±0.04*** | 0.79±0.03 | 0.87±0.02 | 0.87±0.03 | 0.85±0.01 | **0.86±0.02*** |
|  | n-3H | | 0.64±0.05 | 0.57±0.06 | 0.68±0.05 | 0.54±0.03 | 0.63±0.05 | 0.72±0.04* | 0.64±0.04 | **0.74±0.03*** | **0.77±0.02*** | 0.64±0.04 | **0.74±0.03*** | 0.77±0.03 | **0.85±0.**0**2*** | 0.87±0.01 | 0.85±0.01 | 0.86±0.01 |
| F2 adult | | |  |  |  |  |  |  |  |  |  |  |  |  |  |  |  |  |
| Male | n-3D | | 0.60±0.08 | 0.74±0.06 | 0.62±0.07 | 0.71±0.07 | 0.77±0.06 | 0.70±0.06 | 0.79±0.05 | 0.82±0.04 | 0.69±0.06 | 0.79±0.05 | 0.82±0.04 | 0.88±0.03 | 0.89±0.02 | 0.87±0.02 | 0.88±0.03 | 0.92±0.03 |
|  | n-3N | | 0.59±0.08 | 0.72±0.06 | 0.61±0.08 | 0.70±0.06 | 0.76±0.06 | 0.70±0.06 | 0.78±0.05 | 0.82±0.05 | 0.70±0.07 | 0.80±0.05 | 0.83±0.04 | 0.88±0.03 | 0.89±0.03 | 0.87±0.03 | 0.88±0.03 | 0.93±0.01 |
|  | n-3H | | 0.55±0.05 | **0.67±0.04*** | **0.53±0.04*** | **0.64±0.04*** | **0.71±0.04*** | 0.65±0.06 | 0.75±0.03 | 0.78±0.03 | 0.66±0.05 | 0.75±0.03 | 0.79±0.03 | 0.86±0.03 | 0.88±0.03 | 0.86±0.03 | 0.89±0.02 | 0.94±0.01 |
| Female | n-3D | | 0.58±0.04 | 0.69±0.03 | 0.55±0.04 | 0.65±0.04 | 0.73±0.04 | 0.67±0.07 | 0.75±0.03 | 0.78±0.03 | 0.63±0.03 | 0.75±0.03 | 0.77±0.03 | 0.85±0.03 | 0.86±0.02 | 0.85±0.02 | 0.87±0.03 | 0.92±0.02 |
|  | n-3N | | 0.57±0.04 | 0.68±0.05 | 0.54±0.05 | 0.64±0.05 | 0.74±0.07 | 0.65±0.06 | 0.75±0.04 | 0.78±0.04 | 0.67±0.07 | 0.76±0.04 | 0.78±0.06 | 0.85±0.04 | 0.87±0.03 | 0.86±0.03 | 0.87±0.03 | 0.93±0.01 |
|  | n-3H | | 0.56±0.03 | 0.68±0.03 | 0.56±0.02 | 0.64±0.04 | 0.72±0.04 | 0.64±0.03 | 0.75±0.04 | 0.77±0.04 | 0.64±0.04 | 0.75±0.02 | 0.78±0.03 | 0.85±0.02 | 0.86±0.01 | 0.84±0.02 | 0.86±0.02 | 0.91±0.02 |

Genomic DNA isolated from epididymal fat is analyzed for the methylation of CpG sites at the indicated positions in the Lep promoter. The methylation fraction is calculated from the amplitude of cytosine and thymine within each CpG dinucleotide, C/(C+T) *100. The result for each CpG site is represented as the means ± SD. n = 6-8 in each group. *Compared to the n-3 D group, P < 0.05. **#** Compared to the n-3 N group, P < 0.05. n-3 D, n-3 polyunsaturated fatty acids (PUFA) deficient diet; n-3N, normal n-3 PUFA content diet; n-3H, high n-3 PUFA content diet.

**Table 5. Quantitative methylation analysis of the differentially methylated regions in imprinted gene (H19)**

|  |  | CG1 | CG2 | CG3 | CG4 | CG5 | CG6 | CG7 | CG8 | CG9 | CG10 | CG11 | CG12 | CG13 | CG14 | CG15 |
| --- | --- | --- | --- | --- | --- | --- | --- | --- | --- | --- | --- | --- | --- | --- | --- | --- |
| F1 juvenile | |  |  |  |  |  |  |  |  |  |  |  |  |  |  |  |
| Male | n-3D | 0.61±0.03 | 0.61±0.03 | 0.82±0.05 | 0.87±0.04 | 0.91±0.03 | 0.91±0.02 | 0.89±0.03 | 0.84±0.03 | 0.60±0.01 | 0.64±0.02 | 0.59±0.01 | 0.61±0.02 | 0.68±0.03 | 0.66±0..04 | 0.67±0.04 |
|  | n-3N | 0.57±0.02 | **0.55±0.04*** | **0.68±0.04*** | **0.78±0.04*** | **0.85±0.05*** | 0.87±0.05 | 0.85±0.04 | 0.86±0.05 | 0.59±0.02 | 0.63±0.01 | 0.58±0.01 | 0.60±0.01 | 0.70±0.03 | 0.68±0.03 | 0.69±0.04 |
|  | n-3H | 0.60±0.05 | 0.58±0.07 | **0.71±0.08*** | **0.78±0.07*** | 0.87±0.06 | 0.89±0.04 | 0.88±0.04 | **0.89±0.04*** | 0.59±0.02 | 0.63±0.02 | 0.58±0.02 | 0.59±0.02 | 0.71±0.02 | 0.70±0.02 | 0.71±0.03 |
| Female | n-3D | 0.68±0.02 | 0.64±0.02 | 0.75±0.04 | 0.82±0.04 | 0.88±0.03 | 0.91±0.02 | 0.87±0.04 | 0.82±0.05 | 0.58±0.06 | 0.62±0.06 | 0.57±0.06 | 0.59±0.06 | 0.70±0.05 | 0.69±0.05 | 0.71±0.72 |
|  | n-3N | 0.65±0.06 | **0.58±0.04*** | **0.81±0.04*** | 0.86±0.03 | 0.91±0.02 | 0.92±0.01 | 0.89±0.03 | 0.86±0.03 | 0.58±0.02 | 0.62±0.02 | 0.57±0.02 | 0.60±0.01 | 0.72±0.02 | 0.72±0.03 | 0.74±0.03 |
|  | n-3H | **0.58±0.02*** | **0.56±0.01*** | 0.76±0.03 | 0.85±0.03 | 0.90±0.02 | 0.90±0.03 | 0.88±0.03 | 0.88±0.04 | 0.59±0.05 | 0.64±0.05 | 0.58±0.06 | 0.62±0.05 | 0.73±0.02 | 0.74±0.02 | **0.76±0.02*** |
| F1 adult | |  |  |  |  |  |  |  |  |  |  |  |  |  |  |  |
| Male | n-3D | 0.75±0.03 | 0.83±0.03 | 0.89±0.05 | 0.91±0.02 | 0.89±0.04 | 0.91±0.01 | 0.93±0.01 | 0.89±0.02 | 0.53±0.03 | 0.67±0.03 | 0.63±0.04 | 0.67±0.03 | 0.87±0.02 | 0.86±0.03 | 0.88±0.02 |
|  | n-3N | 0.70±0.05 | **0.66±0.03*** | 0.80±0.07 | **0.83±0.05*** | 0.89±0.03 | 0.89±0.02 | **0.90±0.02*** | 0.90±0.02 | 0.52±0.03 | 0.64±0.03 | 0.61±0.03 | 0.66±0.03 | 0.83±0.03 | 0.81±0.03 | 0.85±0.02 |
|  | n-3H | **0.64±0.05*** | **0.71±0.01*** | 0.85±0.03 | **0.86±0.03*** | 0.92±0.03 | 0.88±0.04 | **0.86±0.04*** | 0.86±0.05 | 0.54±0.01 | **0.64±0.01*** | **0.58±0.01*** | **0.62±0.01*** | **0.80±0.02*** | **0.77±0.03*** | **0.81±0.01*** |
| Female | n-3D | 0.66±0.06 | 0.81±0.03 | 0.87±0.03 | 0.87±0.04 | 0.91±0.02 | 0.85±0.04 | 0.86±0.04 | 0.84±0.04 | 0.53±0.03 | 0.62±0.02 | 0.58±0.02 | 0.61±0.01 | 0.77±0.04 | 0.74±0.05 | 0.79±0.04 |
|  | n-3N | 0.65±0.07 | 0.83±0.04 | 0.89±0.02 | 0.89±0.01 | 0.91±0.02 | **0.92±0.01*** | 0.88±0.04 | **0.91±0.01*** | 0.53±0.03 | 0.60±0.02 | 0.56±0.01 | **0.58±0.01*** | **0.73±0.02*** | **0.68±0.02*** | 0.77±0.02 |
|  | n-3H | 0.67±0.04 | 0.79±0.04 | 0.88±0.04 | 0.89±0.04 | 0.93±0.01 | **0.92±0.01*** | 0.88±0.04 | 0.88±0.05 | 0.52±0.02 | **0.59±0.01*** | **0.54±0.01*** | **0.58±0.01*** | **0.73±0.01*** | 0.70±0.01 | 0.78±0.01 |
| F2 adult | |  |  |  |  |  |  |  |  |  |  |  |  |  |  |  |
| Male | n-3D | 0.54±0.05 | 0.60±0.06 | 0.73±0.06 | 0.76±0.05 | 0.88±0.04 | 0.83±0.08 | 0.84±0.04 | 0.84±0.06 | 0.54±0.05 | 0.61±0.02 | 0.54±0.02 | 0.58±0.03 | 0.74±0.04 | 0.74±0.05 | 0.79±0.04 |
|  | n-3N | **0.69±0.06*** | 0.60±0.07 | 0.69±0.05 | 0.74±0.05 | **0.81±0.03*** | 0.82±0.03 | 0.84±0.05 | 0.85±0.03 | 0.57±0.02 | 0.62±0.01 | **0.58±0.01*** | 0.60±0.01 | **0.67±0.01*** | **0.65±0.02*** | **0.69±0.03*** |
|  | n-3H | **0.61±0.03*** | 0.57±0.05 | 0.67±0.05 | **0.71±0.05*** | **0.79±0.06*** | 0.79±0.03 | 0.81±0.03 | 0.84±0.03 | 0.52±0.03 | 0.59±0.01 | 0.53±0.01 | 0.56±0.02 | 0.75±0.04 | 0.75±0.04 | 0.77±0.05 |
| Female | n-3D | 0.66±0.06 | 0.58±0.04 | 0.69±0.08 | 0.73±0.07 | 0.79±0.08 | 0.80±0.07 | 0.81±0.05 | 0.81±0.06 | 0.54±0.04 | 0.62±0.02 | 0.56±0.02 | 0.59±0.03 | 0.77±0.03 | 0.79±0.03 | 0.79±0.02 |
|  | n-3N | **0.59±0.04*** | 0.60±0.07 | 0.67±0.04 | 0.71±0.05 | 0.78±0.06 | 0.79±0.04 | 0.80±0.05 | 0.83±0.04 | 0.53±0.03 | 0.61±0.01 | 0.55±0.01 | 0.57±0.01 | 0.75±0.03 | **0.75±0.03*** | 0.77±0.04 |
|  | n-3H | 0.59±0.06 | **0.65±0.04*** | 0.67±0.03 | 0.74±0.03 | 0.80±0.03 | 0.82±0.02 | 0.81±0.06 | 0.82±0.05 | 0.55±0.03 | 0.61±0.01 | 0.55±0.01 | 0.58±0.01 | 0.74±0.05 | **0.74±0.05*** | 0.77±0.05 |

Genomic DNA isolated from epididymal fat is analyzed for the methylation of CpG sites at the indicated positions in the H19 ICR1. The methylation fraction is calculated from the amplitude of cytosine and thymine within each CpG dinucleotide, C/(C+T) *100. The result for each CpG site is represented as the means ± SD. n = 6-8 in each group. *Compared to the n-3D group, P < 0.05. n-3D, n-3 polyunsaturated fatty acids (PUFA) deficient diet; n-3N, normal n-3 PUFA content diet; n-3H, high n-3 PUFA content diet.

**Table 6. Quantitative methylation analysis of the differentially methylated regions in imprinted gene (Igf2)**

|  |  | CG1 | CG2 | CG3 | CG4 | CG5 | CG6 | CG7 | CG8 | CG9 | CG10 | CG11 | CG12 | CG13 | CG14 | CG15 |
| --- | --- | --- | --- | --- | --- | --- | --- | --- | --- | --- | --- | --- | --- | --- | --- | --- |
| F1 juvenile | |  |  |  |  |  |  |  |  |  |  |  |  |  |  |  |
| Male | n-3D | 0.61±0.03 | 0.76±0.03 | 0.76±0.04 | 0.69±0.04 | 0.81±0.02 | 0.73±0.03 | 0.77±0.02 | 056±0.05 | 0.79±0.03 | 0.73±0.05 | 0.69±0.04 | 0.53±0.06 | 0.68±0.03 | 0.50±0.05 | 0.66±0.05 |
|  | n-3N | 0.61±0.04 | 0.76±0.04 | 0.76±0.04 | 0.69±0.03 | 0.84±0.02 | 0.74±0.02 | 0.80±0.02 | 0.58±0.03 | 0.80±0.02 | 0.71±0.03 | 0.69±0.01 | 0.51±0.03 | 0.66±0.02 | 0.47±0.02 | 0.66±0.02 |
|  | n-3H | 0.59±0.02 | 0.73±0.02 | **0.68±0.06*#** | **0.64±0.01*#** | 0.80±0.02 | **0.67±0.04*#** | 0.74±0.03 | 0.55±0.03 | **0.75±0.03*#** | 0.68±0.02 | 0.65±0.03 | 0.46±0.04 | 0.65±0.01 | **0.43±0.02*** | 0.60±0.03 |
| Female | n-3D | 0.57±0.04 | 0.74±0.03 | 0.73±0.03 | 0.67±0.03 | 0.82±0.04 | 0.74±0.05 | 079±0.04 | 0.58±0.05 | 0.80±0.04 | 0.71±0.03 | 0.68±0.02 | 0.49±003 | 0.66±0.02 | 0.45±0.03 | 0.64±0.04 |
|  | n-3N | 0.57±0.01 | 0.74±0.01 | 0.74±0.02 | 0.66±0.02 | 0.82±0.02 | 0.71±0.03 | 0.79±0.02 | 0.55±0.03 | 0.80±0.02 | 0.70±0.02 | 0.67±0.03 | 0.49±0.04 | 0.64±0.02 | 0.43±0.03 | 0.62±0.02 |
|  | n-3H | 0.57±0.01 | 0.74±0.01 | 0.75±0.01 | 0.67±0.01 | 0.81±0.02 | 0.73±0.03 | 0.79±0.02 | 0.59±0.04 | 0.80±0.02 | 0.69±0.02 | 0.68±0.02 | 0.49±0.04 | 0.65±0.02 | 0.46±0.04 | 0.64±0.04 |
| F1 adult | |  |  |  |  |  |  |  |  |  |  |  |  |  |  |  |
| Male | n-3D | 0.60±0.02 | 0.73±0.02 | 0.74±0.01 | 0.69±0.01 | 0.83±0.02 | 0.73±0.02 | 0.81±0.03 | 0.56±0.03 | 0.81±0.02 | 0.75±0.01 | 0.72±0.03 | 0.47±0.03 | 0.71±0.02 | 0.47±0.04 | 0.65±0.02 |
|  | n-3N | 0.58±0.02 | 0.72±0.03 | 0.73±0.03 | 0.68±0.03 | 0.81±0.03 | 0.71±0.03 | 0.79±0.04 | 0.54±0.03 | 0.79±0.04 | 0.74±0.02 | 0.69±0.04 | 0.44±0.02 | **0.68±0.02*** | 0.45±0.03 | 0.64±0.02 |
|  | n-3H | 0.58±0.02 | 0.74±0.07 | **0.71±0.02*** | 0.68±0.01 | **0.80±0.02*** | 0.71±0.03 | 0.78±0.03 | 0.53±0.03 | 0.79±0.02 | 0.74±0.02 | 0.69±0.04 | 0.45±.02 | **0.68±0.02*** | 0.47±0.04 | 0.62±0.03 |
| Female | n-3D | 0.62±0.04 | 0.72±0.05 | 0.73±0.03 | 0.69±0.04 | 0.84±0.03 | 0.72±0.04 | 0.78±0.03 | 0.49±0.04 | ·0.79±0.03 | 0.72±0.03 | 0.67±0.04 | 0.42±0.04 | 0.64±0.03 | 0.40±0.03 | 0.60±0.05 |
|  | n-3N | 0.59±0.01 | 0.74±0.02 | 0.75±0.01 | 0.70±0.02 | 0.81±0.03 | 0.73±0.05 | 0.78±0.02 | 0.52±0.02 | 0.80±0.02 | 0.73±0.02 | 0.68±0.02 | 0.44±0.01 | 0.65±0.01 | **0.43±0.02*** | 0.61±0.02 |
|  | n-3H | 0.59±0.01 | 0.73±0.01 | 0.73±0.02 | 0.68±0.01 | **0.81±0.01*** | **0.68±0.02*** | **0.74±0.02*#** | 0.47±0.02 | **0.75±0.02*#** | 0.70±0.01 | 0.66±0.01 | 0.42±0.02 | 0.63±0.01 | 0.41±0.0.2 | 0.59±0.02 |
| F2 adult | |  |  |  |  |  |  |  |  |  |  |  |  |  |  |  |
| Male | n-3D | 0.57±0.02 | 0.73±0.02 | 0.73±0.03 | 0.67±0.02 | 0.81±0.03 | 0.72±0.04 | 0.79±0.04 | 0.57±0.05 | 0.80±0.03 | 0.72±0.03 | 0.70±0.03 | 0.46±0.04 | 0.66±0.03 | 0.42±0.04 | 0.61±0.02 |
|  | n-3N | 0.56±0.02 | 0.71±0.01 | 0.72±0.02 | 0.66±0.01 | 0.79±0.01 | 0.71±0.03 | 0.77±0.03 | 0.55±0.03 | 0.79±0.03 | 0.71±0.02 | 0.68±0.02 | 0.44±0.02 | 0.63±0.01 | 0.40±0.03 | 0.60±0.02 |
|  | n-3H | 0.58±0.02 | 0.74±0.02 | 0.76±0.03 | 0.70±0.04 | **0.85±0.03*#** | **0.80±0.03*#** | **0.85±0.02*#** | **0.63±0.05*#** | **0.85±0.02*#** | **0.78±0.02*#** | **0.74±0.03*#** | 0.49±0.04 | 0.68±0.03 | 0.42±0.05 | **0.67±0.04*#** |
| Female | n-3D | 0.59±0.03 | 0.74±0.02 | 0.75±0.02 | 0.69±0.02 | 0.81±0.02 | 0.71±0.03 | 0.77±0.02 | 0.53±0.02 | 0.79±0.02 | 0.71±0.03 | 0.67±0.02 | 0.43±0.01 | 0.62±0.02 | 0.39±0.02 | 0.59±0.03 |
|  | n-3N | **0.65±0.02*** | **0.76±0.01*** | **0.77±0.01*** | 0.70±0.02 | 0.81±0.01 | **0.65±0.03*** | **0.72±0.02*** | **0.47±0.02*** | **0.72±0.02*** | **0.66±0.02*** | **0.62±0.03*** | **0.41±0.02*** | 0.62±0.03 | 0.39±0.02 | 0.58±0.03 |
|  | n-3H | **0.66±0.02*** | **0.77±0.01*** | **0.76±0.01*** | **0.71±0.01*** | 0.81±0.01 | **0.67±0.01*** | **0.73±0.01*** | **0.49±0.02*** | **0.74±0.02*** | **0.67±0.01*** | **0.64±0.01*** | 0.44±0.01 | 0.63±0.02 | 0.41±0.02 | 0.60±0.02 |

Genomic DNA isolated from epididymal fat is analyzed for the methylation of CpG sites at the indicated positions in the Igf2 DMR2. The methylation fraction is calculated from the amplitude of cytosine and thymine within each CpG dinucleotide, C/(C+T) *100. The result for each CpG site is represented as the means ± SD. n = 6-8 in each group. *Compared to the n-3 D group, P < 0.05. **#** Compared to the n-3 N group, P < 0.05. n-3D, n-3 polyunsaturated fatty acids (PUFA) deficient diet; n-3N, normal n-3 PUFA content diet; n-3H, high n-3 PUFA content diet.

**Table 7. Quantitative methylation analysis** **of the differentially methylated regions in imprinted gene (Kcnq1ot1)**

|  |  | CG1 | CG2 | CG3 | CG4 | CG5 | CG6 | CG7 | CG8 | CG9 | CG10 | CG11 | CG12 |
| --- | --- | --- | --- | --- | --- | --- | --- | --- | --- | --- | --- | --- | --- |
| F1 juvenile | |  |  |  |  |  |  |  |  |  |  |  |  |
| Male | n-3D | 0.45±0.03 | 0.46±0.02 | 0.44±0.02 | 0.55±0.03 | 0.54±0.03 | 0.69±0.05 | 0.75±0.05 | 0.86±0.02 | 0.88±0.02 | 0.91±0.01 | 0.89±0.02 | 0.87±0.02 |
|  | n-3N | 0.47±0.02 | 0.47±0.02 | 0.45±0.02 | 0.52±0.02 | 0.51±0.02 | 0.60±0.02 | 0.64±0.03 | 0.88±0.01 | 0.90±0.01 | 0.93±0.01 | 0.91±0.01 | 0.87±0.02 |
|  | n-3H | 0.49±0.03 | 0.50±0.04 | 0.48±0.03 | 0.55±0.02 | 0.54±0.02 | 0.62±0.03 | 0.59±0.06 | 0.84±0.04 | 0.88±0.01 | 0.89±0.02 | 0.88±0.03 | 0.86±0.03 |
| Female | n-3D | 0.47±0.01 | 0.50±0.02 | 0.47±0.01 | 0.56±0.01 | 0.56±0.03 | 0.72±0.03 | 0.75±0.02 | 0.84±0.02 | 0.87±0.02 | 0.90±0.02 | 0.89±0.01 | 0.82±0.07 |
|  | n-3N | 0.47±0.02 | 0.48±0.02 | 0.46±0.01 | **0.54±0.02*** | **0.54±0.02*** | **0.64±0.05*** | **0.66±0.07*** | **0.88±0.01*** | **0.91±0.01*** | **0.93±0.01*** | **0.92±0.02*** | **0.89±0.01*** |
|  | n-3H | 0.47±0.02 | 0.47±0.02 | 0.45±0.03 | **0.52±0.02*** | **0.51±0.02*** | **0.60±0.02*** | **0.62±0.03*** | **0.88±0.01*** | **0.91±0.01*** | **0.93±0.01*** | **0.92±0.01*** | **0.89±0.01*** |
| F1 adult | |  |  |  |  |  |  |  |  |  |  |  |  |
| Male | n-3D | 0.48±0.03 | 0.51±0.03 | 0.49±0.03 | 0.56±0.06 | 0.56±0.06 | 0.67±0.09 | 0.72±0.09 | 0.86±0.02 | 0.89±0.02 | 0.91±0.01 | 0.90±0.02 | 0.89±0.01 |
|  | n-3N | 0.50±0.04 | 0.53±0.04 | 0.51±0.03 | 0.56±0.03 | 0.56±0.02 | 0.63±0.02 | 0.65±0.06 | 0.86±0.01 | 0.89±0.02 | 0.91±0.02 | 0.90±0.02 | 0.90±0.01 |
|  | n-3H | **0.52±0.01*** | **0.56±0.03*** | **0.54±0.03*** | **0.68±0.05*#** | **0.67±0.06*#** | 0.81±0.08 | 0.82±0.07 | **0.79±0.06*#** | 0.85±0.09 | 0.91±0.04 | 0.87±0.04 | **0.85±0.03*#** |
| Female | n-3D | 0.45±0.02 | 0.49±0.03 | 0.47±0.02 | 0.52±0.02 | 0.51±0.02 | 0.60±0.02 | 0.65±0.02 | 0.85±0.01 | 0.88±0.02 | 0.90±0.01 | 0.90±0.01 | 0.89±0.01 |
|  | n-3N | **0.48±0.02*** | 0.50±0.02 | 0.49±0.02 | 0.53±0.01 | 0.53±0.02 | 0.62±0.02 | 0.68±0.05 | 0.87±0.02 | 0.90±0.02 | 0.92±0.02 | 0.91±0.02 | 0.89±0.01 |
|  | n-3H | 0.49±0.05 | 0.51±0.05 | 0.50±0.05 | 0.55±0.05 | 0.55±0.05 | 0.64±0.07 | 0.69±0.08 | 0.85±0.04 | 0.88±0.04 | 0.91±0.03 | 0.89±0.02 | 0.89±0.02 |
| F2 adult | |  |  |  |  |  |  |  |  |  |  |  |  |
| Male | n-3D | 0.56±0.05 | 0.58±0.06 | 0.58±0.06 | 0.70±0.08 | 0.69±0.08 | 0.82±0.05 | 0.86±0.02 | 0.82±0.05 | 0.89±0.02 | 0.89±0.03 | 0.82±0.04 | 0.82±0.07 |
|  | n-3N | **0.50±0.03*** | **0.51±0.04*** | **0.50±0.04*** | **0.55±0.05*** | **0.55±0.05*** | **0.66±0.05*** | **0.71±0.05*** | 0.79±0.04 | **0.81±0.04*** | **0.83±0.05*** | 0.83±0.04 | 0.84±0.02 |
|  | n-3H | 0.53±0.03 | 0.56±0.03 | 0.53±0.03 | **0.59±0.04*** | **0.59±0.04*** | **0.67±0.04*** | **0.71±0.05*** | 0.79±0.02 | **0.80±0.03*** | **0.83±0.02*** | 0.84±0.02 | 0.82±0..04 |
| Female | n-3D | 0.52±0.04 | 0.54±0.04 | 0.52±0.04 | 0.60±0.04 | 0.58±0.04 | 0.66±0.05 | 0.69±0.05 | 0.78±0.04 | 0.80±0.04 | 0.84±0.03 | 0.83±0.03 | 0.82±0.04 |
|  | n-3N | 0.48±0.04 | 0.50±0.04 | 0.48±0.04 | **0.55±0.05*** | 0.54±0.05 | 0.63±0.05 | 0.63±0.06 | 0.79±0.04 | 0.83±0.03 | 0.84±0.04 | 0.83±0.05 | 0.85±0.06 |
|  | n-3H | **0.47±0.02*** | **0.49±0.02*** | **0.48±0.02*** | **0.55±0.02*** | 0.55±0.02 | 0.62±0.03 | **0.62±0.04*** | 0.76±0.04 | 0.77±0.04 | 0.81±0.03 | 0.80±0.04 | 0.85±0.02 |

Genomic DNA isolated from epididymal fat is analyzed for the methylation of CpG sites at the indicated positions in the DMR of Kcnq1ot1 gene. The methylation fraction is calculated from the amplitude of cytosine and thymine within each CpG dinucleotide, C/(C+T) *100. The result for each CpG site is represented as the means ± SD. n = 6-8 in each group. *Compared to the n-3 D group, P < 0.05. **#** Compared to the n-3 N group, P < 0.05. n-3D, n-3 polyunsaturated fatty acids (PUFA) deficient diet; n-3N, normal n-3 PUFA content diet; n-3H, high n-3 PUFA content diet.

**Table 8. Quantitative methylation analysis of the differentially methylated regions in imprinted gene (Plagl1)**

|  |  | CG1 | CG2 | CG3 | CG4 | CG5 | CG6 | CG7 | CG8 | CG9 | CG10 | CG11 | CG12 | CG13 | CG14 | CG15 | CG16 |
| --- | --- | --- | --- | --- | --- | --- | --- | --- | --- | --- | --- | --- | --- | --- | --- | --- | --- |
| F1 juvenile | |  |  |  |  |  |  |  |  |  |  |  |  |  |  |  |  |
| Male | n-3D | 0.52±0.02 | 0.54±0.02 | 0.59±0.02 | 0.61±0.02 | 0.64±0.02 | 0.66±0.02 | 0.71±0.03 | 0.76±0.03 | 0.78±0.02 | 0.76±0.02 | 0.79±0.02 | 0.79±0.02 | 0.79±0.02 | 0.77±0.02 | 0.69±0.02 | 0.70±0.02 |
|  | n-3N | 0.51±0.02 | 0.53±0.02 | 0.59±0.02 | 0.60±0.01 | 0.63±0.02 | 0.65±0.02 | 0.68±0.04 | 0.75±0.05 | 0.77±0.05 | 0.75±0.04 | 0.78±0.02 | 0.78±0.02 | 0.78±0.02 | 0.76±0.01 | 0.69±0.01 | 0.70±0.01 |
|  | n-3H | 0.52±0.01 | 0.54±0.01 | 0.59±0.01 | 0.60±0.01 | 0.63±0.01 | 0.66±0.01 | 0.69±0.01 | 0.75±0.01 | 0.78±0.01 | 0.76±0.01 | 0.79±0.01 | 0.79±0.01 | 0.79±0.01 | 0.76±0.01 | 0.70±0.01 | 0.70±0.01 |
| Female | n-3D | 0.50±0.01 | 0.53±0.01 | 0.58±0.01 | 0.59±0.01 | 0.62±0.01 | 0.63±0.01 | 0.66±0.02 | 0.71±0.02 | 0.74±0.02 | 0.72±0.01 | 0.77±0.01 | 0.76±0.01 | 0.77±0.01 | 0.76±0.01 | 0.69±0.01 | 0.70±03.01 |
|  | n-3N | 0.52±0.02 | 0.54±0.02 | 0.58±0.02 | 0.60±0.03 | 0.63±0.03 | 0.64±0.04 | 0.66±0.06 | 0.71±0.07 | 0.74±0.06 | 0.72±0.05 | 0.75±0.04 | 0.75±0.03 | 0.76±0.03 | 0.74±0.02 | **0.67±0.01*** | **0.68±0.01*** |
|  | n-3H | 0.51±0.02 | 0.54±0.02 | 0.58±0.02 | 0.60±0.01 | 0.62±0.01 | 0.61±0.03 | 0.64±0.02 | 0.69±0.02 | 0.72±0.03 | 0.70±0.02 | 0.75±0.02 | 0.76±0.01 | 0.76±0.02 | 0.75±0.02 | 0.70±0.01 | 0.70±0.01 |
| F1 adult | |  |  |  |  |  |  |  |  |  |  |  |  |  |  |  |  |
| Male | n-3D | 0.47±0.02 | 0.52±0.03 | 0.56±0.03 | 0.60±0.03 | 0.63±0.04 | 0.66±0.04 | 0.71±0.05 | 0.77±0.03 | 0.78±0.02 | 0.76±0.02 | 0.76±0.01 | 0.76±0.01 | 0.76±0.01 | 0.76±0.02 | 0.68±0.01 | 0.68±0.02 |
|  | n-3N | 0.48±0.01 | 0.53±0.01 | 0.56±0.02 | 0.60±0.02 | 0.65±0.03 | 0.67±0.04 | 0.72±0.05 | 0.77±0.04 | 0.79±0.03 | 0.79±0.07 | 0.77±0.01 | 0.79±0.07 | 0.77±0.01 | 0.74±0.01 | 0.68±0.01 | 0.68±0.01 |
|  | n-3H | 0.49±0.04 | 0.55±0.04 | 0.57±0.04 | 0.61±0.04 | 0.67±0.06 | 0.69±0.06 | 0.74±0.07 | 0.78±0.04 | 0.80±0.03 | 0.77±0.02 | 0.77±0.02 | 0.76±0.02 | 0.76±0.02 | 0.74±0.03 | 0.67±0.02 | 0.67±0.02 |
| Female | n-3D | 0.46±0.04 | 0.51±0.03 | 0.55±0.03 | 0.59±0.03 | 0.64±0.04 | 0.67±0.04 | 0.72±0.04 | 0.77±0.03 | 0.79±0.03 | 0.76±0.02 | 0.76±0.02 | 0.76±0.02 | 0.75±0.02 | 0.73±0.02 | 0.66±0.01 | 0.66±0.01 |
|  | n-3N | 0.46±0.02 | 0.51±0.02 | 0.56±0.02 | 0.60±0.02 | 0.65±0.03 | 0.70±0.03 | 0.76±0.02 | 0.80±0.01 | 0.80±0.02 | 0.77±0.01 | 0.77±0.01 | 0.75±0.01 | 0.76±0.01 | 0.73±0.03 | 0.65±0.02 | 0.66±0.02 |
|  | n-3H | 0.43±0.01 | **0.48±0.02*#** | 0.53±0.02 | **0.55±0.01*#** | **0.60±0.01*#** | **0.62±0.03*****#** | **0.66±0.05*#** | **0.72±0.05*#** | **0.75±0.04*#** | **0.72±0.03*#** | 0.74±0.02 | 0.75±0.02 | 0.74±0.02 | 0.72±0.02 | 0.65±0.02 | 0.65±0.02 |
| F2 adult | |  |  |  |  |  |  |  |  |  |  |  |  |  |  |  |  |
| Male | n-3D | 0.58±0.03 | 0.57±0.02 | 0.59±0.01 | 0.62±0.03 | 0.69±0.03 | 0.71±0.05 | 0.77±0.04 | 0.79±0.03 | 0.82±0.02 | 0.78±0.02 | 0.79±0.02 | 0.79±0.01 | 0.79±0.02 | 0.75±0.02 | 0.67±0.02 | 0.68±0.02 |
|  | n-3N | 0.57±0.04 | 0.56±0.03 | 0.58±0.02 | **0.59±0.02*** | **0.66±0.02*** | 0.68±0.04 | 0.74±0.03 | 0.77±0.03 | 0.79±0.03 | 0.78±0.02 | 0.80±0.01 | 0.79±0.01 | 0.79±0.01 | 0.76±0.01 | 0.69±0.01 | 0.69±0.01 |
|  | n-3H | 0.55±0.03 | **0.55±0.02*** | 0.58±0.02 | **0.59±0.02*** | **0.65±0.03*** | 0.68±0.03 | 0.74±0.02 | 0.77±0.04 | 0.80±0.04 | 0.77±0.02 | 0.79±0.02 | 0.79±0.01 | 0.79±0.01 | 0.76±0.01 | 0.68±0.02 | 0.69±0.02 |
| Female | n-3D | 0.56±0.04 | 0.57±0.03 | 0.59±0.02 | 0.59±0.02 | 0.67±0.03 | 0.69±0.03 | 0.74±0.04 | 0.77±0.03 | 0.81±0.03 | 0.78±0.01 | 0.80±0.01 | 0.79±0.01 | 0.80±0.01 | 0.76±0.02 | 0.69±0.02 | 0.70±0.02 |
|  | n-3N | 0.56±0.03 | 0.57±0.02 | 0.60±0.01 | 0.62±0.03 | 0.69±0.04 | 0.71±0.05 | 0.77±0.05 | 0.80±0.04 | 0.83±0.03 | 0.78±0.02 | 0.79±0.01 | 0.81±0.06 | 0.79±0.01 | 0.76±0.02 | 0.68±0.02 | 0.69±0.01 |
|  | n-3H | 0.55±0.03 | 0.56±0.02 | 0.59±0.02 | **0.62±0.02*** | 0.70±0.03 | 0.72±0.05 | 0.77±0.05 | 0.79±0.03 | 0.83±0.02 | 0.79±0.02 | 0.79±0.02 | 0.79±0.02 | 0.79±0.01 | 0.78±0.02 | 0.68±0.02 | 0.69±0.02 |

Genomic DNA isolated from epididymal fat is analyzed for the methylation of CpG sites at the indicated positions in the DMR of Plagl1 gene. The methylation fraction is calculated from the amplitude of cytosine and thymine within each CpG dinucleotide, C/(C+T) *100. The result for each CpG site is represented as the means ± SD. n = 6-8 in each group. *Compared to the n-3D group, P < 0.05. **#** Compared to the n-3 N group, P < 0.05. n-3D, n-3 polyunsaturated fatty acids (PUFA) deficient diet; n-3N, normal n-3 PUFA content diet; n-3H, high n-3 PUFA content diet.


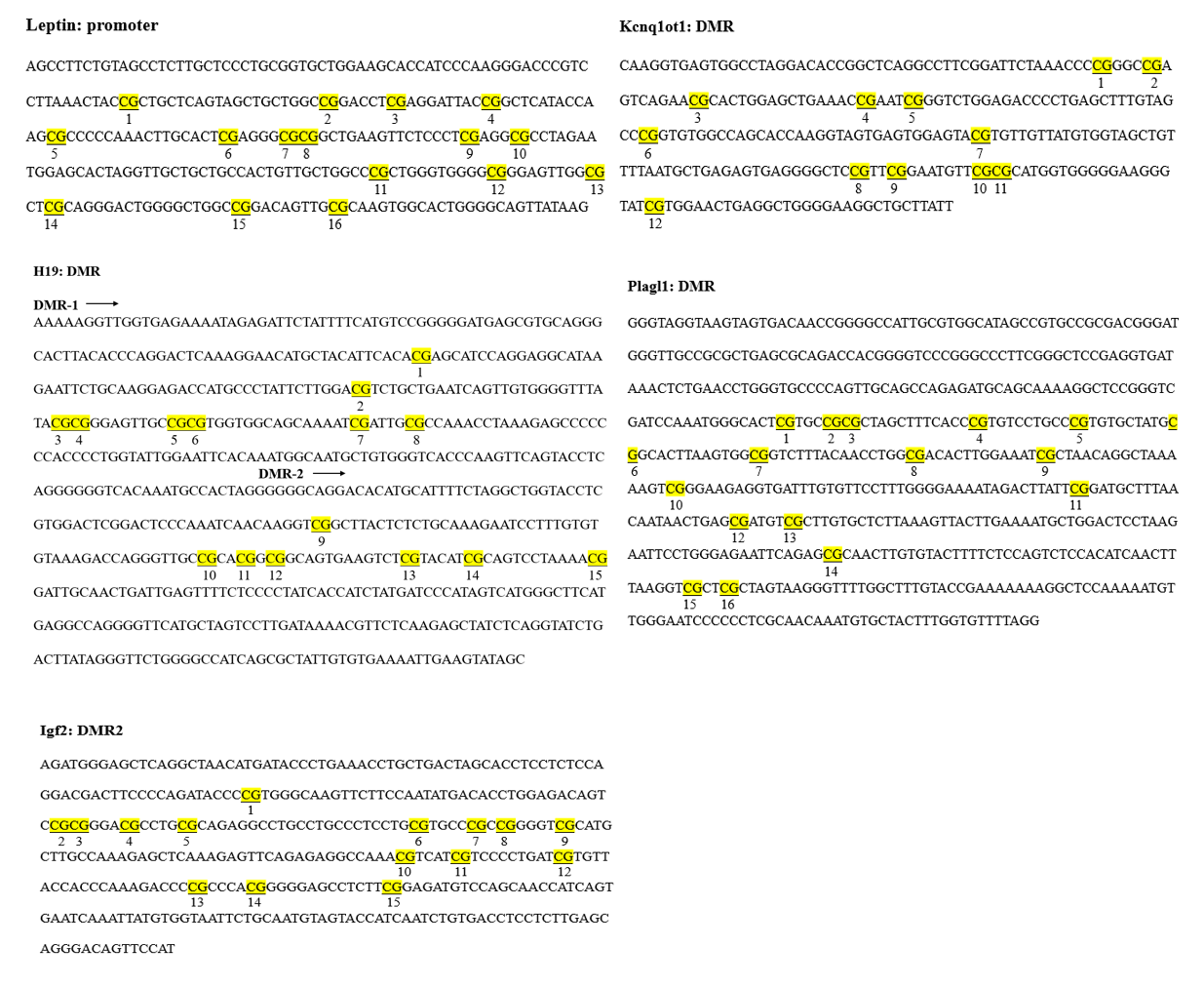


**Figure 1. The promoter region of the leptin and** **differentially methylated regions of imprinted genes (H19, Igf2, Plagl1, kcnq1ot1).** The CG dinucleotides are underlined and numbers were assigned to each of the analyzed CGs. DMR, differentially methylated regions.
